# An Educational Video Game in Trauma Triage at Nontrauma Centers: A Secondary Analysis of a Randomized Clinical Trial

**DOI:** 10.1001/jamanetworkopen.2025.13375

**Published:** 2025-06-04

**Authors:** Deepika Mohan, Baruch Fischhoff, Victor Talisa, Jonathan Elmer, Douglas B. White, Derek C. Angus, Andrew Peitzman, Brad Bendesky, Allyson Cook Chapman, Raquel M. Forsythe, Frank X. Guyette, Allyson M. Hynes, Jonathan J. Oskvarek, Scott D. Weingart, Michael Weinstock, Chung-Chou H. Chang

**Affiliations:** 1 Department of Surgery, University of Pittsburgh School of Medicine, Pittsburgh, Pennsylvania; 2 Department of Critical Care Medicine, University of Pittsburgh School of Medicine, Pittsburgh, Pennsylvania; 3 Department of Engineering and Environmental Policy, Carnegie Mellon University, Pittsburgh, Pennsylvania; 4 Department of Emergency Medicine, University of Pittsburgh School of Medicine, Pittsburgh, Pennsylvania; 5 Department of Neurology, University of Pittsburgh School of Medicine, Pittsburgh, Pennsylvania; 6 Department of Emergency Medicine, Drexel College of Medicine, Philadelphia, Pennsylvania; 7 Department of Surgery, University of California, San Francisco; 8 Department of Surgery, Division of Trauma and Acute Care Surgery, Medical College of Wisconsin, Milwaukee; 9 Department of Emergency Medicine, Medical College of Wisconsin, Milwaukee; 10 US Acute Care Solutions, Canton, Ohio; 11 Department of Emergency Medicine, Summa Health System, Akron, Ohio; 12 Emergency Critical Care, Nassau University Medical Center, East Meadow, New York; 13 Department of Emergency Medicine, Adena Health Systems, Chillicothe, Ohio; 14 Department of Emergency Medicine, Wexner Medical Center at Ohio State University, Columbus

## Abstract

**Importance:**

Understanding how behavioral interventions work and whom they help can increase their effectiveness.

**Objective:**

To evaluate the mechanism of action and heterogeneity of the estimated treatment effect of a customized video game (*Night Shift*) designed to recalibrate physician heuristics (pattern recognition) in trauma triage.

**Design, Setting, and Participants:**

This process evaluation of a randomized clinical trial was performed with a national sample of 800 physicians responsible for the triage of patients with trauma at the emergency departments (EDs) of levels III, IV, and V trauma centers and nontrauma centers in the US. Data were collected online from November 27, 2023, to March 11, 2024. Data were analyzed based on intention to treat.

**Interventions:**

Usual education or customized video game played for 2 hours. All participants completed a virtual simulation, mimicking 3 ED shifts.

**Main Measures and Outcomes:**

The intervention’s mechanism of action was analyzed using signal detection theory, which describes decision-making as the product of perceptual sensitivity (the ability to recognize signal [severe injuries] and noise [minor injuries]) and decisional threshold (tolerance for false-positive or false-negative decisions). The heterogeneity of the estimated treatment effect was evaluated using prespecified subgroup analyses to test moderation by participant characteristics (ie, sex, age, and clinical volume). Findings were validated using a data-driven approach with bayesian additive regression trees.

**Results:**

The 800 participants (566 [71%] male; mean [SD] age, 43.8 [9.4] years) had mean (SD) professional experience of 12.0 (8.4) years, worked at nontrauma centers (488 [61%]) or at level III, IV, or V trauma centers (312 [39%]), and were board-certified in emergency medicine (673 [84%]). Most intervention participants (339 [85%]) played the customized video game for at least 2 hours or until they completed the content, and most (345 of 398 [87%] for the intervention and 231 of 397 [58%] for the control) used the simulation. Assignment to the intervention arm was associated with a reduction in undertriage (22% vs 38%; percentage point difference, 16 [95% CI, 15-18]; *P* < .001). The intervention was associated with a moderate increase in tolerance for false-positive decisions (intervention 0.14 SD units [95% CI, 0.07-0.22]; control 0.53 SD units [95% CI, 0.43-0.63]; Cohen *d* = 0.6) and a small improvement in the ability to recognize severely injured patients (intervention 1.00 SD units [95% CI, 0.94-1.07]; control 0.87 SD units [95% CI, 0.79-0.94]; Cohen *d* = 0.2). Limited heterogeneity of the estimated treatment effect was observed, although participants’ clinical volume was associated with moderation.

**Conclusions:**

In this process evaluation of a randomized clinical trial, exposure to a theory-based video game was associated with liberalized thresholds for transfer and limited heterogeneity of the estimated treatment effect.

**Trial Registration:**

ClinicalTrials.gov Identifier: NCT06063434

## Key Points

**Question:**

How are behavioral interventions targeting physician performance associated with clinical decision-making?

**Findings:**

In this process evaluation of a randomized clinical trial, exposure to an educational trauma triage video game was associated with a moderate increase in the willingness of emergency department physicians to transfer injured patients to trauma centers and a smaller improvement in the recognition of severely injured patients. There was limited heterogeneity of the estimated treatment effect.

**Meaning:**

These findings suggest the potential of educational video games to change physicians’ willingness to adhere to clinical practice guidelines, and therefore may serve as useful adjuncts to existing continuing medical education efforts.

## Teaser

This process evaluation of a randomized clinical trial evaluates the mechanism of action and heterogeneity of the estimated treatment effect of a video game designed to change decision-making heuristics about trauma triage among emergency department physicians.

## Introduction

Across medicine, physicians frequently make diagnostic and therapeutic decisions that diverge from practices recommended by national and international guidelines.^1,2^ While some of the discordance reflects appropriate clinical judgment, at times it represents poor decision-making.^3,4^ Consequently, there is a rapidly growing field of behavioral interventions for health care professionals, ranging from continuing medical education programs to strategies to provide information or skill development support when the user needs to complete a task (“just-in-time” tools) to a wide variety of efforts to influence the architecture of choices.^5,6,7^ Unfortunately, despite enthusiasm for these interventions, there remains a paucity of information about their basic science: how they work and whom they benefit.^8^

Trauma triage is a useful exemplar of a time-sensitive condition where better interventions to increase the implementation of clinical practice guidelines could improve patient outcomes.^9,10,11,12,13^ The American College of Surgeons recommends that physicians treating injured patients at nontrauma centers rapidly screen (ie, triage) them and transfer severely injured patients.^14,15,16^ Despite 4 decades of interventions by stakeholders, undertriage occurs commonly, particularly among patients older than 65 years.^17,18,19,20^ Prior experimental and observational work that included members of our study team^21,22^ suggests that diagnostic error is a major factor in undertriage at nontrauma centers and results in part from physicians relying on their heuristics (defined as mental short cuts or pattern recognition) to identify patients who have severe injuries.

Mohan et al^23,24^ developed a theory-based video game to recalibrate physician heuristics in trauma triage and demonstrated its ability to improve the implementation of guidelines in the laboratory by 11% to 18%. The objective of this study was to evaluate the game’s mechanism of action and heterogeneity of the estimated treatment effect.

## Methods

### Study Overview

A type 1 hybrid effectiveness-implementation trial in the US tested the effect of a novel intervention (a customized video game [*Night Shift*]).^25^ The trial began November 27, 2023, and concluded March 31, 2025. Consistent with the recommendations of the National Institutes of Health and the UK Medical Research Council for developing complex behavioral interventions, we concurrently evaluated the processes by which the intervention affected behavior using qualitative and quantitative methods.^26,27^ Herein, we report the evaluation of the mechanism of action of the intervention, conducted between November 2023 and March 2024. The University of Pittsburgh’s Human Research Protection Office approved the study. All participants provided written informed consent. We followed the Consolidated Standards of Reporting Trials (CONSORT) reporting guideline for randomized clinical trials.

### Trial Participants

We recruited a national convenience sample of physicians performing triage and managing trauma in patients with trauma in the emergency departments (EDs) of levels III, IV, and V trauma centers and nontrauma centers across the US through social media, word-of-mouth, a health care analytics company, and the organizational email distribution lists of several national physician staffing groups between November 27, 2023, and February 7, 2024. We excluded physicians working 50% or more of their time at level I or II trauma centers or only at federal hospitals and those who declined to affirm their willingness to complete all study tasks.

### Study Protocol

The study protocol is found in Supplement 1. Physicians provided informed consent when they enrolled and reported their demographic and professional characteristics. Demographic characteristics included sex (female, male, or prefer not to say) and race and ethnicity, which was included to assess the generalizability of the sample. Participants self-identified as American Indian or Alaska Native, Asian, Black, or White race and Hispanic or non-Hispanic ethnicity or prefer not to say. We randomized eligible participants in a 1:1 allocation ratio, based on a randomization schema generated by our statistician (C.C.H.C.), to receive either the video game intervention or a usual education (passive control) training program. Although we could not maintain blinding after allocation, we masked the assignments until after the completion of data cleaning. Those in the intervention arm received a tablet computer with the game preloaded. We asked them to play the video game for 2 hours (or until they completed the content) within 3 weeks of receipt of their tablet and then to complete an online simulation to assess decision-making. We asked participants in the control arm to complete the same online virtual simulation within 3 weeks of enrollment. Participants in the intervention arm kept their tablet as their honorarium (approximate value $300), while those in the control arm received a $100 gift card conditional on completion of study tasks.

### Interventions

Physicians who rely on heuristics make disposition decisions based on how well patients fit an archetype of severe injury rather than using a rule-based algorithm.^28,29^ We designed the video game to recalibrate heuristics through experience-focused narratives, using feedback from in-game characters on decisions made during the game to highlight the distinction between patients with minimal and severe injury and the noneducational content to increase engagement.^30,31^ We anticipated that exposure to the game would improve recognition of patients with severe injuries and therefore implementation of guidelines. We developed the customized video game originally in 2016, in collaboration with Schell Games.^24,32^ The adventure video game takes approximately 2 hours to complete. We include more details about the development in eMethods in and show a schematic in eFigure 1 in Supplement 2. We did not ask participants in the control arm to complete any supplemental continuing medical education.

### Outcome Assessment

We collaborated with 1st Playable Productions to develop a 2-dimensional virtual simulation that we could use to evaluate physician decision-making in an in silico environment.^33^ The simulation presented users with 36 cases over 45 minutes. We asked users to make decisions as they would in their own environment.

Each case had a 2-dimensional rendering of the patient, a chief symptom, vital signs that updated every 10 seconds, a case history, and a description of the physical examination. Physicians could request diagnostic studies, select from a set of 25 interventions (eg, transfuse blood), or consult a specialist. In the absence of appropriate clinical intervention by the user, severely injured and critically ill patients decompensated and died over the course of the simulated shift. Each case ended when physicians made a disposition decision or the patient died. We include more details about the simulation in eMethods and show a schematic in eFigure 2 in Supplement 2.

### Data Sources and Management

Each physician completed a questionnaire at the time of enrollment providing information about their demographic characteristics (eg, gender, race and ethnicity), practice characteristics, and recent continuing medical education on trauma exposure (eMethods in Supplement 2). The 2024 application of the customized video game uploaded game use statistics (time spent and proportion of content reviewed) to a secure server each time the tablet connected to WiFi. We hosted the simulation on a secure server that captured information about user actions during each case, including time spent, diagnostic studies requested, interventions performed, specialists consulted, and disposition decisions.

### Statistical Analysis

We calculated the response rate as the proportion of physicians who performed any study tasks among those who agreed to participate. We calculated the completion rate as the proportion who completed all assigned tasks. We summarized physician characteristics and use of the intervention.

For the simulation, we summarized the types of decisions made by each physician across the cases. We scored each disposition decision for the trauma cases as adherent or nonadherent with American College of Surgeons clinical practice guidelines.^16^ For patients with severe injuries, we defined compliance as the decision to transfer the patient to a level I or II trauma center. For patients with minor injuries, we defined adherence as a decision to discharge or to admit the patient to the local hospital.

For the outcome analysis, we included anyone who used the virtual simulation for any length of time (ie, those for whom we had any outcome data), regardless of whether they had played the game as instructed (if in the intervention arm), following the intention-to-treat principle. We defined undertriage as the proportion of severely injured patients not transferred to a trauma center and overtriage as the proportion of patients transferred with minor injuries, as specified by the American College of Surgeons.^16^ We used a generalized linear mixed model with logit link function, clustered at the physician level, to estimate the difference in undertriage between physicians in the control and intervention groups. The primary analysis was based on simulated cases of severely injured patients, and the model estimated the probability of an incorrect transfer decision, given the assignment of the physician in the absence of any additional covariates. We also evaluated the dose-response relationship (time spent [duration] to the percentage of game played [amount]) to assess whether a greater dose changed the probability of undertriage. We replicated the analysis to evaluate the association of the intervention with overtriage, estimating the probability of transferred patients having only a minor injury given the assignment of the physician. In sensitivity analyses, we tested the role of nonrandom missingness in our outcome data to explore the potential bias introduced in our effect estimates for undertriage and the role of adjusting for the time spent completing each case during the simulation.^34^

Next, we used a regression-based approach to signal detection theory to evaluate the intervention’s mechanism of action.^35^ Signal detection theory, a method that came to prominence during World War II to improve the performance of radar operators, describes nonadherence with clinical practice guidelines as the product of 2 domains: (1) *perceptual sensitivity* (the ability to distinguish between patients who do and do not meet clinical practice guidelines for transfer) and (2) *decisional thresholds* (the tendency to err on the side of false-positive or false-negative decisions).^36,37^ Perceptual sensitivity reflects physicians’ judgments (both heuristic and analytic) about which patients meet the guidelines for transfer. Decisional thresholds reflect attitudes toward the guidelines. We describe our methods in more detail in eMethods in Supplement 2. To evaluate the magnitude of the effect size, we calculated Cohen *d* as the mean difference between the experimental and comparison condition divided by the pooled SD

Finally, we tested the heterogeneity of the intervention’s association with undertriage, the focus of the behavior change effort, by exploring 3 prespecified subgroups: physician age (<50 or ≥50 years), physician gender (male or female), and clinical workload (<10 or ≥10 shifts/mo).^38,39^ We tested each moderator individually, using mixed-effects regression models with an interaction term. Next, we confirmed our findings with random intercepts bayesian additive regression trees (BART) to model individualized absolute reduction in risk (iARR) of undertriage as a result of playing the customized video game intervention. Estimates and 95% CIs for each iARR were examined using a caterpillar plot, and the associations between variables and iARR estimates were explored using a binary decision tree–based fit-the-fit approach.^40^ eMethods in Supplement 2 provides more details. We used Stata, version 17.0 (StataCorp LLC) to perform the main analysis and the R package dbarts (R, version 4.4.3 [R Core Team]) for BART modeling.

We set the sample size to allow us to test the hypotheses of the parent trial. However, we estimated that if 60% of participants completed the simulation, then using Cohen recommendations for power calculations for behavioral trials with a sample of 800 physicians, we would have the ability to detect small differences (effect size [Cohen *d*] = 0.20) in perceptual sensitivity using a 2-sided hypothesis test, with α = .05 indicating statistical significance and power of 80%.^41^

## Results

### Participant characteristics

We screened 976 physicians and enrolled 800 between November 27, 2023, and February 7, 2024; data collection for this process evaluation ended on March 11, 2024. Four hundred participants were randomized into the intervention and control arms each. Physicians had a mean (SD) age of 43.7 (9.0) years, with a mean (SD) of 12.0 (8.4) years of experience. Two hundred and twenty-six participants (28%) were female, 566 (71%) were male, and 8 (1%) preferred not to say. For racial identity, 3 participants (0.4%) were American Indian or Alaska Native; 116 (15%), Asian; 29 (4%), Black; 587 (73%), White; and 65 (8%), other. Thirty-one participants (4%) identified as Hispanic. Most participants (488 [61%]) worked at nontrauma centers; the remainder (312 [39%]) worked at level III, IV, or V trauma centers. Most (673 [84%]) had board certification in emergency medicine. Almost all (750 [94%]) had completed Advanced Trauma Life Support, the American College of Surgeons course on trauma triage. Additional characteristics are presented in Table 1.

**Table 1.** Participant Characteristics

Characteristic	Participants, No. (%) (N = 800)
Age, mean (SD), y	43.7 (9.0)
Experience, mean (SD), y	12.0 (8.4)
Sex	
Female	226 (28)
Male	566 (71)
Prefer not to say	8 (1)
Race	
American Indian or Alaska Native	3 (0.4)
Asian	116 (15)
Black	29 (4)
White	587 (74)
Prefer not to say	65 (8)
Ethnicity	
Hispanic	31 (4)
Non-Hispanic	725 (91)
Prefer not to say	44 (6)
Type of residency	
Emergency medicine	673 (84)
Family practice	94 (12)
Internal medicine	25 (3)
General surgery	3 (0.4)
Prefer not to say	5 (1)
Have not completed a fellowship,	710 (89)
Completed ATLS	750 (94)
Time since completed ATLS^a^	
<1 y	89 (12)
1-4 y	305 (42)
>4 y	334 (46)
Completed the American Board of Emergency Medicine resuscitation module	113 (14)
Region of country of employment	
Northeast	131 (16)
Southeast	266 (33)
Midwest	121 (15)
Southwest	145 (18)
West	137 (17)
Trauma center designation of hospital of primary (≥50%) employment	
Level III	166 (21)
Level IV	138 (17)
Level V	8 (1)
Nontrauma center	488 (61)
Never work at a level I or II trauma center	514 (64)
No. of shifts worked per month, mean (SD)	13 (3.6)
Play video games for fun	463 (58)
How did you learn about the trial	
Email invitation from physician staffing agency	607 (76)
Email invitation from health care analytics company	7 (1)
Social media	99 (12)
Friends	87 (11)

Abbreviation: ATLS, Advanced Trauma Life Support.

^a^ Of the 750 physicians who completed the ATLS, 22 did not affirm this; the denominator is therefore 728.

**Figure 1.**
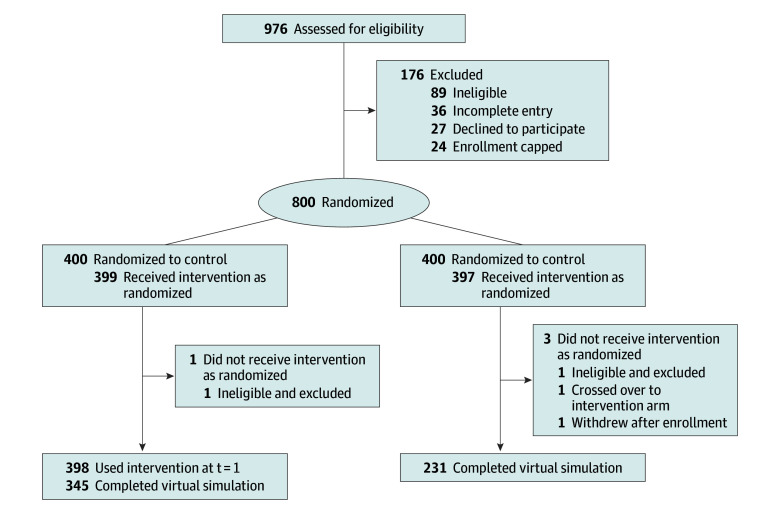
Sampling Frame for the Process Evaluation Description of enrollment, allocation, and follow-up to evaluate the intervention’s mechanism of action and heterogeneity of estimated treatment effect. *t* = 1 indicates postenrollment.

A total of 339 physicians in the intervention arm (85%) played the video game for at least 2 hours and/or completed entire game (eTable 1 in Supplement 2). Among the participants who received the allocated intervention, 345 of 398 (87%) in the intervention arm and 231 of 397 (58%) in the control arm used the outcome assessment tool (Figure 1).

### Outcome Assessment

The intervention was associated with a moderate increase in tolerance for false-positive decisions (intervention 0.14 SD units [95% CI, 0.07-0.22]; control 0.53 SD units [95% CI, 0.43-0.63]; Cohen *d* = 0.6) and a small improvement in the ability to recognize severely injured patients (intervention 1.00 SD units [95% CI, 0.94-1.07]; control 0.87 SD units [95% CI, 0.79-0.94]; Cohen *d* = 0.2). Findings are detailed as follows.

#### Descriptive Summary of Simulation Responses

Physicians completed a mean (SD) of 30.5 (5.4) cases, spending a mean (SD) of 2.7 (1.9) minutes reading and responding to each case. They made a mean (SD) of 4.7 (1.9) decisions in each trauma case. They ordered diagnostic tests in a mean (SD) of 21.4 (4.3) cases, performed an intervention in a mean (SD) of 16.7 (5.6) cases, and consulted a specialist in a mean (SD) of 5.0 (5.3) cases.

#### Physician Performance

**Figure 2.**
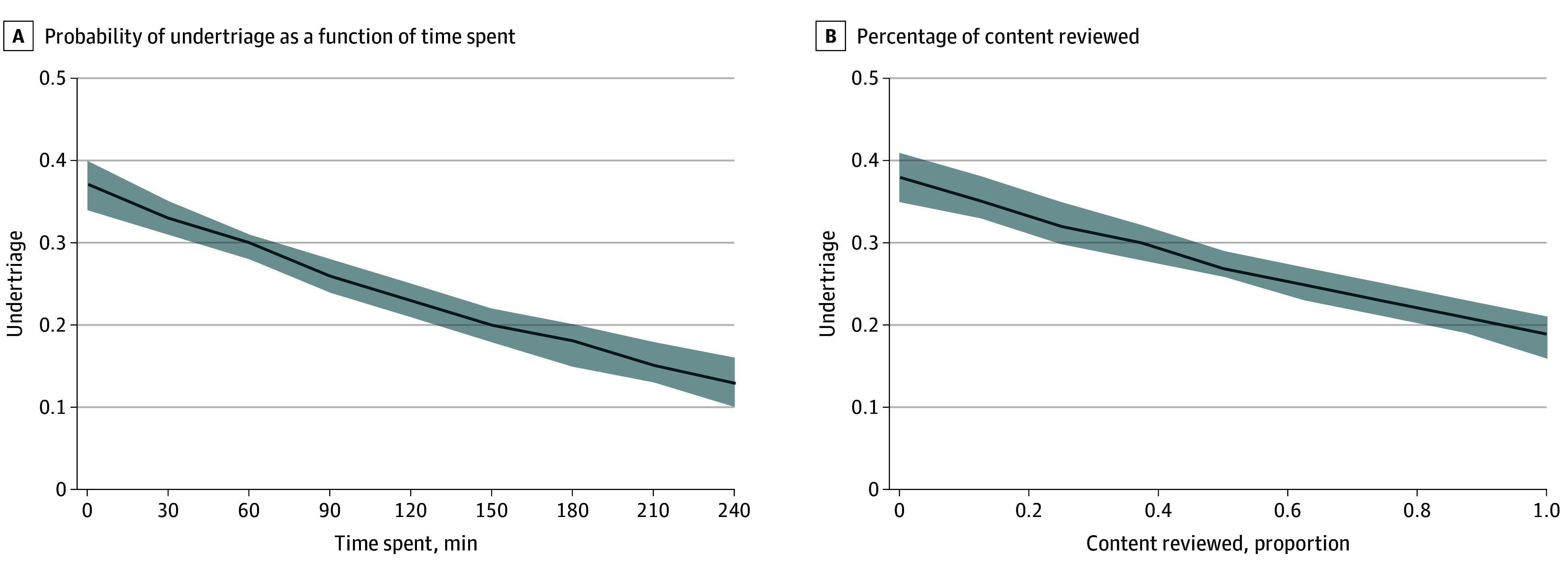
Dose-Response Relationship We asked physicians to play the game for 2 hours or until they completed all the content (whichever came first). We show the probability of undertriage as a function of time spent (A) and the percentage of content reviewed (B). Shaded areas represent 95% CIs.

Assignment to the intervention arm was associated with a reduction in undertriage (22% [intervention] vs 38% [control]; percentage point difference, 16 [95% CI, 15-18]; *P* < .001) as shown in Table 2. As shown in Figure 2, we noted an association between dose and undertriage. We also observed an association between assignment and overtriage (39% [intervention] vs 34% [control]; percentage point difference, 5 [95% CI, 4-7]; *P* < .001). Sensitivity analyses to explore the potential bias introduced by nonrandom missingness (eTable 2 in Supplement 2) and to adjust for the estimated effect of time spent on simulation cases did not alter our conclusions.

**Table 2.** Estimates of the Probability of Outcomes Derived From Mixed Effects Regression Models

Model	Estimations, % (95% CI)			*P* value^a^
	Control group	Intervention group	Difference	
Model 1: benefit of intervention				
Undertriage	38 (34-42)	22 (19-24)	16 (15-18)	<.001
Model 2: harm of intervention				
Overtriage	34 (30-38)	39 (35-43)	5 (4-7)	<.001
Model 3: moderator of the benefit of the intervention: age				
Undertriage if <50 y	37 (44-41)	21 (19-24)	16 (14-16)	.27^a^
Undertriage if ≥50 y	44 (36-52)	22 (17-27)	22 (19-25)	
Model 4: moderator of the benefit of the intervention: sex				
Undertriage if male	41 (37-46)	23 (20-26)	18 (17-20)	.48^a^
Undertriage if female	31 (25-37)	18 (14-22)	13 (11-15)	
Model 5: moderator of the benefit of the intervention: clinical volume				
Undertriage if worked ≥10 shifts/mo	40 (36-44)	22 (19-24)	18 (17-20)	.05^a^
Undertriage if worked <10 shifts/mo	27 (18-35)	21 (15-27)	6 (3-8)	

^a^ Represents the significance of the interaction between the moderator and the intervention.

#### Mechanism of Action

**Figure 3.**
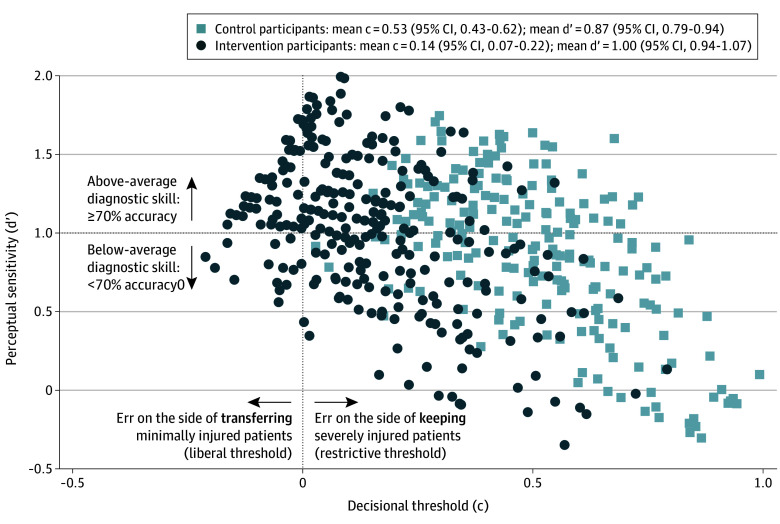
Trial Physicians’ Decisional Thresholds and Perceptual Sensitivity Physicians in the intervention arm had a lower decisional threshold for transfer and a higher perceptual sensitivity than those in the control arm.

Assignment to the intervention arm was associated with a higher perceptual sensitivity (for intervention, 1.00 [95% CI, 0.94-1.07] SD units; for control, 0.87 [95% CI, 0.79-0.94] SD units; *P* < .001). This difference indicates a small (0.20) improvement in the recognition of severely injured cases. Assignment to the intervention arm was also associated with a more liberal decisional threshold (for intervention, 0.14 [95% CI, 0.07-0.22] SD units; for control, 0.53 [95% CI, 0.43-0.62] SD units; *P* < .001). This difference indicates a moderate (0.60) increase in willingness to transfer. We show the estimates of individual physicians’ signal detection theory parameters in Figure 3.

#### Heterogeneity of Estimated Treatment Effect

In the initial test of the heterogeneity of the interventions’ estimated treatment effect, only the participant’s clinical workload (ie, number of shifts worked per month) was associated with moderation as shown in Table 2. Participants who worked 10 or more shifts per month and were in the intervention arm had a significant reduction in their undertriage compared with those in the control arm (control group, 40% vs intervention group, 22%; percentage point difference, 18; 95% CI, 17-20; *P* = .05). In contrast, the intervention and control participants who worked less than 10 shifts per month had similar performance (control group, 27% vs intervention group, 21%; percentage point difference, 6; 95% CI, 3-8). We confirmed this finding using data-driven approaches (eFigure 3 in Supplement 2). However, these results also demonstrated that the amount of heterogeneity was small (eFigures 4 and 5 in Supplement 2).

## Discussion

To improve our understanding of how to develop effective behavioral interventions for health care professionals, we conducted a process evaluation of a randomized clinical trial testing a video game intervention. Although the game changed behavior, it did not act as we had expected. Exposure to the game was associated with a small improvement in physicians’ recognition of severely injured patients, the intended mechanism of action, but with a larger change in physicians’ willingness to transfer patients. There was an association between exposure to the intervention and benefit among all participants.

### Strengths and Limitations

The present study has several strengths. First, we assembled a large nationally representative, diverse cohort of ED physicians, supporting the precision and generalizability of the conclusions.^42^ Using a combination of social media, word of mouth, and partnership with physician practices across the country, we reached physicians working at about 40% of all EDs in the country.^43^ Second, it replicates the results from prior efforts to evaluate the estimated effect of the video game on physician performance, using smaller convenience samples of physicians.^23,24^ The concordance of the effect size provides confidence in the rigor of the results. Third, we identified an association between the dose of the intervention and its estimated effect. Conceptually, this evidence confirms observations from a systematic review that contact time is associated with outcomes, although the mechanism remains unclear.^44^

The signal detection theory evaluation provides insights that can inform knowledge translation. We designed the game to recalibrate physician heuristics, adopting the principle of narrative persuasion from the preventive health literature.^45^ As players progressed through the game, they not only encountered didactic information about relevant contextual cues when evaluating patients with trauma but also experienced the emotional repercussions of errors of judgment. We anticipated that the behavior change techniques and game mechanics would manifest as an improvement in perceptual sensitivity. Instead they produced a greater effect on decisional thresholds, which explains the unintended increase in overtriage. The clinical importance of this outcome is unclear, since the American College of Surgeons defines a well-functioning region as one that achieves less than 5% undertriage, even if that requires up to 50% overtriage.^16^ However, researchers interested in the development of theory-based implementation strategies in other contexts may benefit from our insights into how narrative persuasion affects behavior.^46^

Second, we tested the presence of heterogeneity in the estimated treatment effect. A study can demonstrate an overall mean benefit for an intervention when it provides a large benefit in a subset and none for the majority.^47^ Understanding the characteristics of both groups facilitates efforts to disseminate the intervention more precisely. Notable findings included limited heterogeneity, although the number of shifts worked appeared to moderate the association between the game and undertriage. One explanation is that busier physicians have less time to review clinical practice guidelines, allowing greater potential for improvement. Alternatively, these physicians may work at institutions that implicitly or explicitly encourage their health care professionals to retain more severely injured patients.^18,48,49^ The intervention may have convinced them to contravene these norms.

The study had several limitations. First, we used a simulation to conduct this process evaluation because low base rates of injury preclude precise estimates of physicians’ cognitive processes in practice.^50^ Second, physicians exposed to the intervention may have had an unfair advantage when completing the virtual simulation. However, different companies designed the 2 products. In addition, both groups of physicians reviewed the same tutorial before beginning the simulation, further reducing any learning effects. Third, differences in response rates between the intervention and control groups, likely secondary to the perceived differences in the material value of the honoraria, may have introduced bias.^51^ However, during sensitivity analyses, the estimated effect of the intervention persisted. Fourth, our sample size was too small to conduct reliable inferences quantifying the robustness of our findings in the flexible BART-based models. Instead, we used BART to confirm the findings from our prespecified subgroup analyses.

## Conclusions

The results of this secondary analysis of a randomized clinical trial suggest that educational adventure video games have the potential to improve physician performance in time-sensitive conditions and appear to act by increasing physicians’ willingness to implement clinical practice guidelines. The limited heterogeneity of the estimated treatment effect suggests most physicians will benefit from exposure to the intervention, informing the design of future efforts to distribute the intervention.

## Notes

Supplement 1.
Trial Protocol

Supplement 2. **eMethods.** Intervention and Simulation Development, Physician Survey, Intention-to-Treat Principle, Signal Detection Theory Analysis, and Heterogeneity of Treatment Effect Analyses **eTable 1.** Physician Use of the Intervention **eTable 2.** Sensitivity Analysis to Test the Effect of Missing Outcome Data Using Inverse Probability Weighting **eFigure 1.** Schematic of the Conceptual Framework of the Intervention **eFigure 2.** Schematic of the Conceptual Framework of the Simulation **eFigure 3.** Association Among Clinical Workload, Gender, and Individualized Absolute Risk Reduction Estimates **eFigure 4.** Caterpillar Plot of individualized Absolute Risk Reduction Estimates From Bayesian Additive Regression Trees With 3 Predictors (Primary Model) **eFigure 5**. Caterpillar Plot of Individualized Absolute Risk Reduction Estimates From Bayesian Additive Regression Trees With 14 Predictors (Secondary Model)

Supplement 3.
Data Sharing Statement
