# Supplementary material for: An Educational Video Game in Trauma Triage at Nontrauma Centers: A Secondary Analysis of a Randomized Clinical Trial
Source: JAMA Netw Open. 2025 Jun 4;8(6):e2513375. doi: 10.1001/jamanetworkopen.2025.13375 (PMC12138726; doi:10.1001/jamanetworkopen.2025.13375)
Supplement: Supplement 1. — Trial Protocol [file jamanetwopen-e2513375-s001.pdf]

PittPRO Pitt Protocol Review Online

Date: Monday, April 28, 2025 8:29:56 AM Print Close

View: Pitt SF: Basic Study Information 8.2

Basic Study Information

1. * **Title of study:**
   Using video games to increase implementation of clinical practice guidelines in trauma triage

2. * **Short title:**
   D & I Trial of Night Shift

3. * **Brief description:**
   Two-thirds of older adults with severe injuries are treated at non-trauma centers (under-triage), despite evidence that care at trauma centers reduces preventable deaths (lives that appropriate and timely medical care would have saved). One of the critical barriers to reducing preventable deaths after injury is the absence of interventions that improve physician decision making. Our objective is to test the real-world efficacy of a video game intervention designed to promote physician use of evidence-based practices in trauma triage.

4. * **What kind of study is this?**
   Single-site study

5. * **Will an external IRB act as the IRB of record for this study?**
   ○ Yes ● **No**

6. * **Local principal investigator:**
   Deepika Mohan

   * **Is this your first submission, as PI, to the Pitt IRB?**
   ○ Yes ● **No**

7. * **Does the local principal investigator have a financial interest related to this research?**
   ○ Yes ● **No**

8. **Attach the protocol:**
   - Sponsor/Multicenter/Investigator-initiated protocol
   - Coordinating Center supplement
   - Emergency Use Consent/ Protocol/ FDA Form 3926

- Exempt Application form

Document	Category	Date Modified	Document History

There are no items to display

View: Pitt SF: Funding Sources (not integrated with Grants)

Funding Sources

1. * **Indicate all sources of support:**
External funding

2. * **Identify each organization supplying funding for the study:**

Funding Source	Sponsor's Funding ID	Grants Office ID	Attachments	Pitt Awardee	Grant Recipient
National Institutes of Health			Compiled application 2.24.pdf	yes	Deepika Mohan, MD

View: Pitt SF: Study Team Members

Study Team Members

1. * **Identify each person involved in the design, conduct, or reporting of the research:**

Name	Roles	Department/ School	Affiliation	Involved in Consent	Qualifications	Financial Interest
Derek Angus	Co-investigator	U of Pgh | School of Medicine | Critical Care Medicine	Pitt faculty	no	Dr. Angus is chair of the Critical Care Medicine Department and is an internationally recognized clinical researcher. Dr. Angus is a leader in develo... view all	no
Jacqueline Barnes	Key Personnel / Support Staff	U of Pgh | School of Medicine | Critical Care Medicine	Pitt staff	no	Ms. Barnes is a member of the Qualitative Core, who is trained to perform semi-structured interviews. She will perform half the interviews with trial... view all	no
Chung-Chou Chang	Co-investigator	U of Pgh | School of Medicine | Medicine	Pitt faculty	no	Chung-Chou Ho (Joyce) Chang, PhD will serve as a Co-Investigator. Dr. Chang is a Professor of Statistics with expertise in adaptive clinical trials, ... view all	no
Jonathan Elmer	Co-investigator	U of Pgh | School of Medicine | Emergency Medicine	Pitt faculty	no	Jonathan Elmer, MD MS will serve as a Co-Investigator. Dr. Elmer is an assistant professor of Emergency Medicine, who spends half his clinical time i... view all	no
Kenneth Hunter	Key Personnel /	U of Pgh | School of	Pitt staff	yes	Kenneth Hunter is a Qualitative	no

Name	Roles	Department/ School	Affiliation	Involved in Consent	Qualifications	Financial Interest
	Support Staff	Nursing | Acute and Tertiary Care			Research Coordinator who will assist with the study interview process. Kenneth has a Bachelors in Natural Sciences fr... view all	
Deepika Mohan	Principal Investigator	U of Pgh | School of Medicine | Critical Care Medicine	Pitt faculty	yes	Deepika Mohan, MD will be the Principal Investigator. Dr. Mohan is an Associate Professor of Critical Care Medicine and Surgery, who has over a decad... view all	no
Kimberly Rak	Key Personnel / Support Staff	U of Pgh | School of Medicine | Critical Care Medicine	Pitt staff	no	Dr. Rak runs the Qualitative Core in the Department of Critical Care Medicine, established to facilitate the collection of high-quality qualitative ... view all	no
Dan Ricketts	Key Personnel / Support Staff	U of Pgh | School of Education | Office of the Dean	Pitt staff	no	Mr. Ricketts is the Director of Research Information Technology for the Health Sciences within the Senior Vice Chancellor's office at Pitt. He will ... view all	no
Mary Ryabik	Primary Study Coordinator	U of Pgh | School of Medicine | Critical Care Medicine	Pitt staff	yes	As project manager, Mary Beth will be coordinating tasks to drive the study to on-time completion, and within budget. Mary Beth has a strong backgrou... view all	no

Name	Roles	Department/ School	Affiliation	Involved in Consent	Qualifications	Financial Interest
Ali Smith	Key Personnel / Support Staff	U of Pgh | School of Medicine | Critical Care Medicine	Pitt staff	no	Oversees and assigns roles for research administrative staff	no
Douglas White	Co-investigator	U of Pgh | School of Medicine | Critical Care Medicine	Pitt faculty	no	Douglas White, MD will serve as a Co-Investigator. Dr. White is a Professor of Critical Care Medicine, and a world-renowned health services research... view all	no

2. **External team member information: (Address all study team members in item 1. above and leave this section blank)**

Name	Description
There are no items to display	

3. **Have you,** Deepika Mohan**, verified that all members of the research team have the appropriate expertise, credentials, training, and if applicable, child clearances and/or hospital privileges to perform those research procedures that are their responsibility as outlined in the IRB application?**

* ● **Yes** ○ No

View: Pitt SF: Study Scope 8.1

Study Scope

Check all that apply

1. * Will this study actively recruit any of the following populations?

- [ ] Adults with impaired decision-making capacity
- [ ] Children (under the applicable law of the jurisdiction in which the research will be conducted (<18 years for PA))
- [ ] Children who are Wards of the State
- [x] Employees of the University of Pittsburgh/UPMC
- [ ] Medical Students of University of Pittsburgh as primary research group
- [ ] Students of the University of Pittsburgh
- [ ] Neonates of uncertain viability
- [ ] Non-viable neonates
- [ ] Non-English speakers
- [ ] Nursing home patients in the state of Pennsylvania
- [ ] Pregnant women
- [ ] Prisoners
- [ ] N/A

2. * Will any Waivers be requested?

- [ ] Waiver/Alteration of Consent
- [ ] Waiver to Document Consent
- [x] Waiver/Alteration of HIPAA
- [ ] Exception from consent for emergency research
- [ ] N/A

3. * Will this study involve any of the following?

- [ ] Specimens
- [ ] Honest Broker to provide data/specimens
- [ ] Return of Results to Subjects or Others
- [ ] Fetal tissue
- [x] N/A

4. * Will Protected Health Information be collected?

- [ ] Pitt medical records
- [ ] UPMC medical records
- [x] Other Institutions' medical records
- [ ] N/A

5. * Other Requests?

- [ ] Deception (if not Exempt, also requires Waiver/Alteration of Consent)
- [ ] Emergency Use / Single Patient Expanded Access (using FDA Form 3926)
- [ ] Placebo Arm

- [ ] Withdraw from usual care
- [x] N/A

6. * **Determining Scientific Review:**
Received External funding where scientific merit was established as a condition of funding

7. * **Has this study (or substantially similar study) been previously disapproved by the Pitt IRB or, to your knowledge, by any other IRB?**
○ Yes ● **No**

Review the HRPO policy, if participating in research at the VA Pittsburgh Healthcare System or using funding from the VA

8. * **Does the study use an approved drug or biologic, use an unapproved drug or biologic, or use a food or dietary supplement to prevent, diagnose, cure, treat, or mitigate a disease or condition?**
○ Yes ● **No**

9. * **Does the study evaluate the safety or effectiveness of a device (includes in-vitro laboratory assays)?**
○ Yes ● **No**

10. * **Is this application being submitted to convert an approved study from OSIRIS to PittPRO?** (Tip Sheet)
○ Yes ● **No**

11. * **Does your research protocol involve the evaluation or use of procedures that emit ionizing radiation and, after reviewing this HUSC guidance, does your research protocol require HUSC review? (If yes, upload the HUSC form in the Local Supporting Documents section). If you are unsure of review requirement, select yes.**
○ Yes ● **No**

View: Pitt SF: Research Sites

Research Sites

1. **Choose all sites that apply:**
   University of Pittsburgh

   * **Select the University of Pittsburgh sites where research will be conducted:**
   Main Campus – Pittsburgh

   List university owned off-campus research sites if applicable:

2. **Describe the availability of resources and the adequacy of the facilities to conduct this study:**
   The PI has a private office (~200 ft.) within the Department of Surgery, Presbyterian hospital with standard office furnishings. The University of Pittsburgh provides secretarial and fiscal staffs to all investigators at no direct cost. The PI has a PC and a MacBook Pro laptop computer. The Department of Surgery and Critical Care Medicine has several black and white printers and scanners. Software includes Microsoft Office and STATA 17.0 for statistical analysis.

   Intellectual resources available at the University of Pittsburgh related to this project, and within walking distance of the offices of the PI include the Center for Research on Health Care and the Clinical Research Investigation and Systems Model of Acute Illness Center, each with their own seminar series and faculty network for research development, feedback, and dissemination.

View: Pitt SF: Local Site Documents

Click **Continue** as this page was intentionally left blank.

View: Pitt SF: Recruitment Methods

Recruitment Methods

* **Will you be recruiting individuals for participation in this study?**
● **Yes** ○ No

1. * **Describe who will be recruiting individuals for participation for this study:**
The Principal Investigator and study Project Manager.

2. * **Select all methods to be used for recruitment:**
Email/Listserv/Electronic Mailing List
Letters sent to potential participants
Pitt+Me
Telephone scripts

3. * **Provide details on your recruitment methods:**
For the intervention and control groups, we will be utilizing 3 national physician staffing groups (ApolloMD, US Acute Care Solutions (USACS), UPP EM Partners) to recruit 800 physicians via email. The email will describe the trial, provide access to an eligibility survey and consent form. 20 physicians who are selected via this method will also be asked via email to participate in qualitative interviews at 1 and 6 months after enrollment in the study.

ED directors, ED physicians, Trauma directors and Paramedics - we will purchase an email distribution list from IQVIA. IQVIA has a list of >30,000 eligible healthcare professionals (e.g., ED physicians), and can provide a random sample on request. Based on their experience, 5% of recipients will agree to participate in a research study for which they receive a wage-based honorarium. We therefore anticipate emailing 1600
healthcare professionals with invitations to participate in our study. The email will describe the trial, provide access to an eligibility survey and consent form.

Patients/Surrogates - Pitt+Me will be used to recruit patients and/or surrogates. An initial screening of the registry identified 14,000 eligible patients (age over 65; sustained an injury). We will contact a purposive sample (based on sex, geographic location, and race/ethnicity) to identify 20 people willing to discuss their experiences during a 30-60 minute semi-structured interview. Call Center staff interview interested participants by telephone using pre-screening questions customized to the specific study and then refer eligible participants to the study team. We will contact potential participants via phone call, letter or email.

4. * **Describe all compensation/incentives offered to participants and timing of these offers:**
Participants in the intervention group will receive an iPad (estimated cost $350/

device) on which to play the video game (Night Shift 2024), which they will keep as their honorarium. The iPads will be mailed in December 2023. In addition, participants will be compensated for playing three booster doses at three month intervals in the amount of $25.00 per 20-minute booster dose via a gift card. Separately, 20 participants in this group will be asked to complete qualitative interviews at the 1 month and 6 month intervals after enrollment. They will be compensated at a rate of $100 per hour to participant in a 30-60 minute interview via a gift card.

Participants in the control group will receive $100/hour spent on completing study tasks which will take approximately one hour. The tasks include completing a web-based online assessment tool and questionnaire. Participants will be paid via gift card after verification that they have completed the assessment tool.

Decision Makers (ED physicians, ED Directors, Trauma Directors) - these participants will be compensated at $100 per hour for participation in one qualitative interview that will be 30-60 minutes in length. They will be compensated at the completion of their interview via gift card.

Paramedics - these participants will be compensated at $100 per hour for participation in one qualitative interview that will be 30-60 minutes in length. They will be compensated at the completion of their interview via gift card.

Patients and Surrogates - these participants will be compensated at $100 per hour for participation in one qualitative interview that will be 30-60 minutes in length. They will be compensated at the completion of their interview via gift card.

5. **Recruitment materials:** (attach all material to be seen or heard by subjects, including advertisements and scripts)

	Document	Category	Date Modified	Document History
View	Pitt + Me Website Wording(0.01)	Recruitment Materials	2/20/2024	History
View	Recruitment Email-Interview-ED-Trauma Director - First Responder.docx(0.02)	Recruitment Materials	9/13/2023	History
View	Recruitment Email-Interview-MDs already in Study.docx(0.02)	Recruitment Materials	9/13/2023	History
View	Recruitment Email-Study-MD Staffing Firms.docx(0.02)	Recruitment Materials	9/13/2023	History
View	Recruitment Script - Interview - Patient-Surrogate.docx(0.02)	Recruitment Materials	9/13/2023	History

View: Pitt SF: Study Aims

Study Aims

1. * **Describe the purpose, specific aims, or objectives and state the hypotheses to be tested:**

Transfer of severely injured patients to trauma centers, either directly from the field or after evaluation at non-trauma centers, reduces preventable morbidity and mortality. Failure to transfer these patients appropriately (i.e., under-triage) remains common, and occurs in part because physicians at non-trauma centers make diagnostic errors when evaluating the severity of patients' injuries. We developed Night Shift, a theory-based adventure video game, to recalibrate physician heuristics (intuitive judgments) in trauma triage and established its efficacy in the laboratory. This is a Type 1 hybrid effectiveness-implementation trial to determine whether the game changes physician triage decisions in real-life, and hypothesize that it will reduce the proportion of patients under-triaged.

We will recruit 800 physicians who work in the emergency departments (EDs) of non-trauma centers in the US, and will randomize them to the game (intervention) or to usual care (control). We will ask those in the intervention group to play Night Shift for 2 hours within 2 weeks of enrollment and again for 20 minutes at quarterly intervals. Those in the control group will receive only usual care (i.e., nothing supplemental). The primary outcome is the proportion of severely injured patients older than 65 years, treated by trial participants, triaged concordantly with clinical practice guidelines, assessed using Medicare claims data. Secondary outcomes include 30-day mortality and readmissions (composite outcome), and functional independence. We will evaluate contextual factors influencing reach, adoption, implementation, and maintenance with interviews of a subset of trial participants (n=20) and of other key decision makers (e.g., patients, first responders, administrators [n=100]).

The hypotheses to be tested are as follows:

Primary (behavioral analysis) - The proportion of patients under-triaged by physicians in the game-based training group will be lower than the proportion of patients under-triaged by those in the usual care group.

Secondary (outcome analysis) - The proportion of patients with adverse patient-centered outcomes (i.e., 30-day mortality and readmissions, loss of functional independence) will be lower in the game-based training group than in the usual care group.

Secondary (outcome analysis) - The proportion of patients over-triaged by physicians in the game-based training group will be equivalent to the proportion over-triaged by physicians in the usual care group

Secondary (mediation) - Under-triage will mediate the effect of the intervention on patient-centered outcomes.

Exploratory (heterogeneity of treatment effect) -

[1] The intervention will have the same effect on males as on female trial participants.
[2] The intervention will have the same effect on White compared as on non-White trial participants.
[3] The intervention will have a greater effect on trial participants who expressed positive attitudes to game-based learning before enrollment compared to those who do not have positive attitudes.
[4] Trial participants in the game-based training group who use Night Shift 2024 for greater than 2 hours will under-triage a smaller proportion of severely injured patients than those who use the game for less than 2 hours.
[5] The proportion of patients under-triaged by trial participants will be lower immediately after exposure to the intervention (i.e., in the first 30 days) compared to late post-exposure period (i.e., 30-89 days).
[6] Trial participants with greater parameters of compliance on SONAR (the experimental tool to measure determinants of physicians' decision making) will under-triage a smaller proportion of severely injured patients than those with lower parameters of compliance.

2. * **Describe the relevant prior experience and gaps in current knowledge including preliminary data. Provide for the scientific or scholarly background for, rationale for, and significance of the research based on existing literature and how it will add to existing knowledge:**

In prior work, we exploited insights from the behavioral science literature to develop novel interventions to recalibrate physician heuristics in trauma triage.* Specifically, we identified theories of behavior change from other domains (e.g., threat detection) and applied them to trauma triage. We delivered these interventions as video games to increase engagement and to facilitate distribution. In pilot trials, physicians who played the games made 10-18% fewer errors on a validated virtual simulation compared with those who completed a gold-standard, text-based educational program, an effect that persisted through the 6-month follow-up. These data underscore the potential for our interventions to change behavior significantly and durably. The objective of our research, the next logical step in the effort to achieve the long-term goal of reducing the burden of injury on older adults, is to test the effect of the novel interventions on behavior in practice. Our overarching hypothesis is that exposure to our intervention will increase implementation of clinical practice guidelines, which will reduce the incidence of preventable morbidity and mortality after injury.

*NB: For citations for the above information see attached grant application.

View: Pitt SF: Study Design

Study Design

1. **Total number of subjects to be enrolled at this site (enter -1 for chart reviews, banking, registries):**
   880

2. **Describe and explain the study design:**
   Randomized clinical trial with participants randomized to either the intervention group or to a control group.

3. **Describe the primary and secondary study endpoints:**
   Primary end-point: under-triage of severely injured patients treated by trial participants.
   Second end-points: over-triage of severely injured patients treated by trial participants; 30-day mortality and 30-day readmission of severely injured patients (composite end-point) treated by trial participants; functional independence of severely injured patients, treated by trial participants, at discharge among those with independence prior to the injury.

4. **Provide a description of the following study timelines:**

   Duration of an individual subject's active participation:
   For participants in the intervention group: 12 months
   Interview subjects: two weeks

   Duration anticipated to enroll all subjects:
   Intervention/Control arm: 3-4 weeks
   Interview Arm: 3-4 weeks

   Estimated date for the investigator to complete this study (complete primary analyses):
   10/1/2025

5. **List the inclusion criteria:**
   Intervention/Control: We plan to recruit board-certified physicians who work exclusively in the EDs of non-trauma centers in the US, and who triage adult trauma patients as part of their practice.

   Interviews:
   -ED directors at non-trauma centers, trauma directors at Level I/II trauma centers, first responders (paramedics)
   -Patients (65 and older) and/or surrogates who were recently treated at a non-trauma center for a traumatic injury using Pitt+Me

Medicare beneficiaries (65 and older) who were treated by study physicians, with an Inpatient or Outpatient claim filed between 2023-2024 in which one of the discharge diagnoses is associated with a moderate to severe injury. We will provide ResDac with a validated crosswalk between ICD10-CM diagnoses codes and the severity of injury to identify the relevant patients.

6. **List the exclusion criteria:**

We plan to exclude non-physician healthcare professionals (e.g., nurse practitioners, physician assistants) because of variation in billing practices (e.g. some bill under their own identifiers while others do not) that will confound outcome assessment. We will also exclude physicians who work at both trauma and non-trauma centers (because this limits the number of eligible patients they encounter), and those who work outside the continental US (because of differences in referral patterns).

7. **Will children or any gender, racial or ethnic subgroups be explicitly excluded from participation?**

● **Yes** ○ No

* **Identify the subgroups and provide a justification:**

We are studying physicians and other care providers (ED/Trauma directors) who treat or care for an adult patient population.

8. **Describe the power analysis used and cite your method of statistical analysis. If a power analysis is not possible, thoroughly justify the sample size required for the study, including appropriate literature citation (alternatively provide page reference in attached protocol):**

Based on prior studies, we assume that we can link ≥75% of trial participants to their National Provider Identifiers (NPIs), allowing us to link trial data to claims records, that each physician will evaluate a median of 1-2 eligible patients in the trial period, and that the intraclass correlation coefficient is 0.45. Under these assumptions, with 400 physicians per group (N=800), we can detect between a 7.4–10% difference in under-triage between the intervention and control groups with 80% power and a significance level of 0.05, using a one-sided hypothesis test. We have chosen a one-sided hypothesis test to boost statistical efficiency and because we prioritize identification of a positive effect of the intervention. A negative effect would produce the same outcome as a null: the decision not to proceed with a Phase IV trial. Parameters for the various quantities included in our primary analysis are quite uncertain, making precise sample size simulation impossible. This difference was estimated using a simple comparison of proportions and is thus conservative. By adjusting for multiple sources of variance in our final analysis, our actual power is expected to be greater.

View: Pitt SF: Research Activities

Research Activities

1. * **Provide a detailed description of all research activities (including screening and follow-up procedures) that will be performed for the purpose of this research study. This description of activities should be complete and of sufficient detail to permit an assessment of associated risks.**

Intervention and Control:
We have partnered with 3 national physician staffing groups. Cumulatively, these groups employ approximately 4500 physicians, cover ≥30 states, staff ≥600 emergency departments (EDs), and provide care to ≥16 million patients each year. We will ask physician leaders of the organizations, with which we have partnered, to distribute an email to their staff that describes the trial, and includes a link to the consent form. Physicians who provide consent will then receive a survey that collects demographic data which will allow us to assess eligibility.

Qualified physicians will be randomized with an allocation ratio of 1:1, based on a randomization schema generated by our statistician (CCC) in STATA 17.0 (Statacorp, TX) with blocks of 4 or 8.6. Trial coordinators will link the randomization schema to the enrollment data, and will assign participants to groups. Although we cannot maintain blinding after allocation, our data analysts will not have access to that information.

We will pre-load new iPads with Night Shift 2024 and will mail the devices to those allocated to the intervention group. We will ask participants to spend a minimum of two hours playing Night Shift 2024 within 2 weeks of receiving their device, and then return to the game for 20 minutes at 3, 6, and 9 months. Participants will keep their iPad as a fixed honorarium (approximate value: $350), and will also receive a conditional monetary honorarium if they complete any of the booster doses that they complete ($25/session). We will issue three email reminders and a phone call during the first two weeks after enrollment, and then quarterly reminders during the subsequent trial period. Participants have the option of not completing the assigned study task, but we do not have pre-specified criteria for discontinuing or modifying the allocated intervention.

Participants in the usual care group will complete outcome assessment tools, and will receive a conditional, wage-based honorarium ($100/hour spent) upon completion of study tasks.

Interviews:
We will interview a subset of physicians in the game arm (n=20) at one and six months after enrollment, to learn about their experiences with the game and contextual modifiers to adoption, implementation (anticipated/actual), and maintenance (anticipated/actual) of guideline-concordant trauma triage. We will supplement this data with interviews of a national sample of other key decision-makers (i.e., patients, ED directors at non-trauma centers, trauma directors at Level I/II trauma centers, first responders [n=80]). We will focus on barriers and facilitators of implementation of clinical practice guidelines in trauma triage (anticipated [month

1] and actual [month 6]) using an interview guide developed using the Consolidated Framework for Implementation Research (CFIR).19 We include the interview guide in the attachments.

Hospitals:
We will obtain information about the organizational characteristics of each hospital at which physicians work using the 2022 Centers for Medicare and Medicaid Services (CMS) Healthcare Cost Report Information System (HCRIS). HCRIS contains facility-level characteristics of all non-federal hospitals, including geographic location (state, region), participation in a hospital network, total bed count, ICU bed count, ownership, and teaching status. Since HCRIS does not contain data on the trauma center status of hospitals, we will link HCRIS to the Trauma Information Exchange Program (TIEP) to identify the trauma center designation for each hospital in 2023.

Patients:
We will use ResDac to obtain Inpatient, Outpatient, and Professional Claims filed with Medicare Fee-for-Service (FFS) and Advantage for beneficiaries older than 65 years old with ICD10 codes associated with injuries for 2023-2024. Data elements abstracted directly from the claims will include patient demographics, the hospital identifier, date of admission/discharge, ICD10 diagnosis/procedure codes, disposition status (e.g., home, nursing home), and vital status (date of death). We will map ICD10 diagnosis codes to abbreviated injury scores (AIS) using a well-validated program (ICDPIC), and will calculate injury severity scores (ISS). We will also estimate the presence or absence of serious illness and organ failure using validated algorithms. Finally, we will estimate functional status pre-injury by conducting a 90-day lookback from the date of admission to identify claims filed at skilled nursing or rehabilitation facilities.

We will identify patients treated by trial physicians by linking the names of trial participants to NPIs, and searching for claims filed by those physicians in the Inpatient, Outpatient, and Professional Claims files. We will then construct episodes of care for each patient by linking Outpatient and Inpatient Standard Analytic files to identify visits to acute care, non-federal hospitals. We will order claims by day and classify visits that occur within one day of each other as part of a single episode of care. For episodes with multiple claims from the same day, we will order the claims under the assumption that patients will move from non-trauma centers to trauma centers, and from low-volume hospitals to high-volume hospitals.

Data management:
Data sources include consent forms collected electronically, survey data collected electronically, audio files and transcripts of interviews, and claims files purchased from the Centers of Medicare Services (CMS). All data will be stored on a secure server at the University of Pittsburgh. Data integrity checks will be conducted periodically every 6 to 12 months by the principal investigator and the security team for the University of Pittsburgh School of Medicine Information Technology department. Additional processes to promote data quality will include range checks for data values, and analysis by two different statisticians.

Confidentiality:
We will create a linkage file that connects personal data with anonymized identifiers, and will then will use de-identified data for all analyses. This file will be encrypted and stored on our secure server, and only the study team will have access to it.

2. Upload a copy of all materials used to collect data about subjects: (Attach all surveys, interview/focus group scripts, and data collection forms except for case report forms, SCID or KSADS):

	Document	Category	Date Modified	Document History
View	Questions-Demographics for Participants.docx(0.01)	Data Collection	9/1/2023	History
View	R01 Nightshift Paramedic Interview Guide_ 08 25 2023_accepted.docx(0.01)	Data Collection	9/1/2023	History
View	Instrument to assess fidelity of intervention - Month 6.docx(0.01)	Data Collection	8/30/2023	History
View	Instrument to assess fidelity of intervention delivery and receipt - Month 1.docx(0.01)	Data Collection	8/30/2023	History
View	R01 Nightshift_SDM Interview Guide_ 08 25 2023.docx(0.01)	Data Collection	8/30/2023	History
View	R01 Nightshift_Patients Interview Guide_ 08 25 2023.docx(0.01)	Data Collection	8/30/2023	History
View	R01 Nightshift Trauma Director Interview Guide_ 08 25 2023.docx(0.01)	Data Collection	8/30/2023	History
View	R01 Nightshift MD Interview Guide_ 08 25 2023.docx(0.01)	Data Collection	8/30/2023	History
View	R01 Nightshift ED Director Interview Guide_ 08 25 2023_accepted.docx(0.01)	Data Collection	8/30/2023	History

3. * Will blood samples be obtained for research purposes?

○ Yes ● **No**

View: Pitt SF: Consent Process

Consent Process

Enter N/A in response to the following questions if a Waiver of Consent is requested for all research activities or if no subjects are being enrolled.

1. * **Indicate where the consent process will take place and at what point consent will be obtained:**
   As part of the email invitation to participate in the study, recipients will have the option of reviewing a consent form on-line and subsequently providing demographic information. Eligible subjects will receive further information about study tasks. The physician, director and first responder interview groups will occur in the same fashion. The patient/surrogate groups will be screened via phone by Pitt+Me and consented per their recommendation using phone/email/letter.

2. * **Describe the steps that will be taken to minimize coercion and undue influence, including assurance that there is sufficient time for subjects to make an informed decision:**
   Participants will be given three days to respond to the email, at which point they will receive a reminder. Those who do not respond within 1 week to the recruitment email will not be contacted further. To minimize coercion, the Qualtrics document will emphasize that participation is voluntary, and consent can be withdrawn at any point during the study.

   Interview participants will be asked if they understand the process and wish to proceed with the interview. They will be reminded that they can withdraw from participation at any time.

   Contact information for the PI and study coordinator are provided to participants and they can contact us at any time with questions or concerns.

3. **For studies that involve multiple visits, describe the process to ensure ongoing consent:**
   Participants have the option at any time to pull out of the study and the information that was collected will be used up until that point. Participants completing the booster session have the option not to complete the booster sessions. Participants completing in more than one interview have the option not to complete subsequent interviews. Interview participants will be asked if they understand the process and wish to proceed with the interview. They will be reminded that they can withdraw from participation at any time.

4. * **Steps to be taken to ensure the subjects' understanding:**
   We will ensure that all the documentation is checked for clarity and readability prior to contacting trial participants. When recruiting participants our documentation will specify that: 1) we are recruiting people for an NIH-funded study; 2) participation is voluntary; 3) they will receive no direct benefit from participation; 4) they will receive an honorarium for their time; 5) they will be given a time commitment for

participation; 6) they may withdraw from the study at any time. We will also provide contact information for the PI, which they can use to gain further details about the trial. During interview sessions, study personnel will confirm participant's understanding of the study and the requirements and the option to withdraw at any time.

5. * **Are you requesting an exception to the IRB policy related to the informed consent process:**

○ Yes ● **No**

View: Pitt SF: Consent Forms

Consent Forms

1. **Consent Forms:**

	Document	Category	Date Modified	Document History
View	Consent via Qualtrics- ED MDs-ED Directors-Trauma Directors-wo-demo_Version_0.01.docx(0.02)	Consent Form	9/13/2023	History
View	Consent via Qualtrics-Physician Interviews_Version_0.01 (1).docx(0.02)	Consent Form	9/13/2023	History
View	Consent via Qualtrics-Patient or Surrogate-wo-demo_Version_0.01.docx(0.02)	Consent Form	9/13/2023	History
View	Consent via Qualtrics- Intervention and Control-wo demo_Version_0.02 (2).docx(0.03)	Consent Form	9/13/2023	History

Refer to the following templates and instructional documents:

- Guidance - Consent Wording
- Template - Consent Document - Short Form
- HRP-090 - SOP - Informed Consent Process for Research
- HRP-091 - SOP - Written Documentation of Consent

View: Pitt SF: Waiver/Alteration of HIPAA Authorization

Waiver/Alteration of HIPAA Authorization

The use or disclosure of PHI involves no more than minimal risk to the privacy of individuals, based on, at least, the presence of the following elements.

1. * **Describe your plan to protect the identifiers from improper use or disclosure:**

   We will apply to ResDac for permission to obtain Medicare claims data. The identifiable information that will be included in this dataset will be the patient's date of birth and zip code. All other identifiers are removed. ResDac requires the suppression of cells with sizes less than 10 to prevent identification of individuals within small zip codes. All Medicare claims data will be stored on the HSRDC, which has stringent protocols in place to prevent improper use or downloading of the claims files.

2. * **Describe your plan to destroy the identifiers at the earliest opportunity consistent with conduct of the research, unless there is a health or research justification for retaining the identifiers or such retention is otherwise required by the law:**

   Upon completion of the research project (completion of all analyses and publication of manuscripts), we plan to destroy the data and send written certification of the destruction of the files to CMS within 30 days.

3. * **Describe written assurances that the PHI will not be reused or disclosed to any other person or entity, except as required by law, for authorized oversight of the research project, or for other research for which the use or disclosure of PHI would be permitted by this subpart:**

   We will not reuse or disclose any PHI to any person or entity, except as required by law and as to complete the objectives of this research project or other research for which the use of PHI would be permitted.

4. * **Indicate why the research could not be practicably be conducted without the waiver or alteration:**

   Obtaining consent from Medicare patients to use their records is not practical given the large numbers of patients, and the fact that many will be deceased by the time we acquire their records.

5. * **Indicate why the research could not practicably be conducted without access to and use of the PHI:**

   It is not possible to conduct this research without access to and the use of protected health information. In particular we could not study the triage of trauma patients across the country without access to patient's ZIP codes. Additionally, we are specifically interested in the practice patterns of physicians when treating older adults, which in turn requires access to data about patients' age.

6. * **Explain why the nature and amount of the medical record information to be**

collected is felt to be the minimum necessary to conduct this research study:

Privacy analysts at ResDAc ensure that researchers receive the minimal amount of PHI necessary to answer the research question. In this case, we need the patient's zip code and age. Other identifying information (social security number, name, etc) will not be included in the data files provide to the research team.

View: Pitt SF: Medical Records

Medical Records

1. * **Describe what protected health information will be obtained from a non-UPMC/Pitt covered entity for research purposes and how the HIPAA requirements will be met:**

   We ask ResDac to send us Medicare part A and part B claims for patients with injuries (based on ICD10 codes) in 2024. These claims include the age of patients and their home zip codes. We are applying for a waiver of HIPAA Authorization.

View: Pitt SF: Electronic Data Management

Electronic Data Management

1. * **Will only anonymous data be collected (select NO if identifiers will be recorded at anytime during the conduct of the study)?**

○ Yes ● **No**

Select all identifiers to be collected during any phase of the research including screening:

**Name:**	☑	**Internet Protocol (IP) Address:**	☑
**E-mail address:**	☑	**Web Universal Resource Locators (URLs):**	☐
**Social security #:**	☐	**Social security # (for Vincent payment only):**	☐
**Phone/Fax #:**	☑	**Full face photo images or comparable images:**	☑
**Account #:**	☐	**Health plan beneficiary #:**	☐
**Medical record #:**	☐	**Device identifiers/serial numbers:**	☐
**Certificate/license #:**	☐	**Vehicle identifiers/serial #/license plate #:**	☐
		**Biometric identifiers, finger and voice prints:**	☐

* **a: Will you be collecting any of the following location data: geographic subdivisions smaller than a State such as street address, city, county, precinct, zip, geocodes, etc.?** ● **Yes** ○ No

* **b: Will you be collecting any date information such as birth date, death, admission, discharge, date of surgery/service?** ● **Yes** ○ No

c: List any other unique identifying numbers, characteristics or codes related to an individual that are to be collected:

Physician will provide name and email address on enrollment, and will be assigned an identifier. All information collected from the App and website will be under that identifier. Additional identifiers collected on the website will include: age, sex, race, ethnicity, board certification, certification in ATLS, year certification received, NPI numbers.

Medicare claims data as

previously stated. Age, sex, race and location data will be provided but with masked identifiers.

d: Will you be collecting any data subject to the General Data Protection Regulation (GDPR)? ○ Yes ● No

---

* **For ALL identifiable data collected, will you be coding the data by removing the identifiers and assigning a unique study ID/code to protect the identity of the participant?**	● Yes ○ No
* **Will the data be HIPAA de-identified?**	● Yes ○ No
* **Briefly describe your plan to store coded data separately from the identifiable data:**	All trial participants will be assigned a unique study identifier at the time of enrollment, and all data associated with that individual will use that identifier. Information linking the data codes with subject identities will be stored separately from the recorded data. At no time will we reveal subject identities in any description or publication of the research for scientific purposes.

2. **During this study, will restricted data as defined by the University's Data Risk Classification matrix (https://www.technology.pitt.edu/security/data-risk-classification-and-compliance) be processed, stored, or transmitted?**
   ● Yes ○ No

3. * **During this study, will sensitive data (https://www.hrpo.pitt.edu/electronic-data-security) be collected where disclosure of identifying information could have adverse consequences for subjects or damage their financial standing, employability, insurability, educational advancement, reputation or place them at risk for criminal or civil liability?**
   ● Yes ○ No

4. * **Select all locations where data will be stored or archived(including e.g., personal / employer laptop or desktop): If you have access to University owned or controlled resources, facilities, or repositories, such as computer servers, please choose that option to comply with the Research Data Management Interim Policy R1 14.**

   Please note that to address Research Security Requirements, University data must be stored in University owned, controlled, or approved repositories, such as Pitt OneDrive. If UPMC or external electronic repositories must be used, they must be approved by Pitt IT.

Storage Device	Description	Identifiable Data	Sensitive Data	De-Identified/ Anonymous

					Data
View	Other: Personal desktop, laptop, or other device	Home Apple Desktop - Kim Rak	yes	no	no
View	PITT: OneDrive / Sharepoint	Critical Care Medicine Sharepoint Site	yes	no	yes
View	Server: Pitt Department Managed Server	Data will be downloaded from the study website on to the HSRDC server and will be made available to the study team. Medicare claims data is also being obtained and will be stored on the HSRDC.	yes	no	yes
View	PITT owned desktop, laptop or other device	Pitt Qualitative Research laptop* will be used to upload information from recorder to SharePoint *PC Laptop with Bitlocker, which by default, uses the AES encryption algorithm in cipher block chaining (CBC) or XTS mode with a 128-bit or 256-bit key. University of Pittsburgh managed Antivirus.	no	no	no
View	Other: Cloud Storage Service other than Pitt or UPMC OneDrive	Pitt Zoom	yes	no	yes

5. * Select all technologies being used to collect data or interact with subjects: Technologies selected in this section may require a Vendor Security Risk Assessment, which can be requested here.

Mobile App
Text messaging
Electronic audio, photographic, or video recording or conferencing
Web-based site, survey, or other tool

6. * Mobile App – identify all mobile applications, including text messaging apps, used to collect data during any phase of the research:

name	Identifiable
Night Shift 2024	yes

7. * Text Messaging - Identify all uses of SMS / cellular text messaging:

name	Identifiable

name	Identifiable
Text Message	no

8. * Video, Audio, Images – identify all uses of video, audio, photography, etc. to be used to collect data during any phase of the research:

	name	Identifiable
View	Electronic audio recording	yes
View	Pitt Zoom	yes

9. * Web Based Technologies – identify all web based technologies to be used to collect data during any phase of the research:

	name	Identifiable
View	Website	yes
View	Pitt Licensed Qualtrics	

View: Pitt SF: Data Safety and Monitoring

Data Safety and Monitoring

1. * **Describe your plan to periodically evaluate the data collected regarding both harms and benefits to determine whether subjects remain safe. The plan might include establishing a data monitoring committee and a plan for reporting data monitoring committee findings to the IRB and the sponsor:**
   The research team will meet monthly over the course of the study to discuss subject recruitment, study procedures, and data analysis. During these meetings there will be ongoing review of study procedures to ensure the privacy of research subjects and the confidentiality of their data have not been violated. We will review data during these meetings, and will ensure their validity and integrity. We will discuss any change to the anticipated benefit-to-risk ratio of study. Any adverse events, including breach of confidentiality, will be immediately reported to the University of Pittsburgh IRB. In addition, the funding institute is establishing a data safety monitoring board that will oversee trial progress and will meet annually.

2. * **Describe your plan for sharing data and/or specimens:**
   As per the funding institute's requirements, we plan to share data by using a data repository. We will upload de-identified data sets, data dictionaries and meta-data to openICPSR upon completion of trial milestones (e.g., completion of primary analysis and reporting of results).

3. **If any research data is collected, stored, or shared in a paper format, address what precautions will be used to maintain the confidentiality of the data:**
   Not applicable.

View: Pitt SF: Risk and Benefits

Risk and Benefits

1. * Enter all reasonably foreseeable risks, discomforts, hazards, or inconveniences to the subjects related to subjects' participation in the research:

View

Research Activity	Use of video game and on-line assessment
Common Risks	None
Infrequent Risks	Lack of voluntariness, employability, confidentiality
Other Risks	None.

2. * Describe the steps that will be taken to prevent or to minimize risks:

Voluntariness: We will make it very explicit that research participation is not a condition of employment by the different staffing organizations. However, there may yet be a perception of lack of voluntariness. The Office of Human Subjects Research Protection writes: "Employees are likely to view their employers as authority figures to whom they must show deference, which could undermine the freedom of their choice." We will therefore separate research participation from employer purview, asking the chief medical officers of the company (who will distribute our email invitation) to make it clear that they will not receive any information about study progress from the investigative team.

Confidentiality: a compromise of the security of trial results would result in a breach of confidentiality and violation of privacy. We estimate this risk to be less than 1 in 100. Strict confidentiality and privacy procedures have been put in place to protect against any potential public disclosure of individual provider performance data. We will use physician identifiers (e.g., names and UPIN numbers linked to unique study IDs) for all data that we collect. All physician-level data will be de-identified for analysis and reporting.

Employability: Study participation is not a condition of employment. Theoretically, if physicians demonstrate poor triage performance, and employers learn this information, it might affect their desire to retain the physician. However, given our safeguards to protect the privacy of trial participants, and the time lag between participation and our acquisition of claims data, we estimate this risk to be less than 1 in 1000.

3. Financial risks - will the subject or insurer be charged for any research required procedures?

○ Yes ● **No**

4. Describe the steps that will be taken to protect subjects' privacy:

To protect participants' privacy we will take the following steps:

1. Zoom sessions will be conducted in private rooms.

2. We will not collect any sensitive information, since none is required for completion of the study aims.
3. All study subjects will be assigned unique study identifiers that will appear on all data collection instruments, documents, and files used in the statistical analysis and manuscript preparation.
4. Only limited team members will have access to personal information needed for tracking and informed consent. No personal information concerning study participants will be released without their written consent.

5. **What steps will be taken in the event that a clinically significant, unexpected disease or condition is identified during the conduct of the study:**
Not applicable.

6. **Describe the potential benefit that individual subjects may experience from taking part in the research or indicate if there is no direct benefit. Do not include benefits to society or others:**
There is no direct benefit.

7. **Do you anticipate any circumstances under which subjects might be withdrawn from the research without their consent?**
○ Yes ● **No**

8. **Describe procedures that will be followed when subjects withdraw from the research, including partial withdrawal from procedures with continued data collection and data already collected:**
If participants decide to withdraw from the study, all data will continue to be used up to the point of withdrawal unless they request that we destroy it. There is no penalty for refusing to participate or withdrawing.

View: Pitt SF: Conflict of Interest Ver2

Conflict of Interest

Institutional Financial Interests

1. * **To the best of your knowledge, has the University of Pittsburgh optioned or licensed technology that will be tested or evaluated in this research?**
   ○ Yes ● **No**

View: Pitt SF: Ancillary Reviews

Ancillary Reviews

1. **Ancillary reviews or notifications selected below are required based on previous answers to questions. If a selection is incorrect, return to the appropriate page and adjust the answers to questions on that page:**

- [ ] Conflict of Interest (**COI**)
- [ ] Clinical and Translational Research Center (**CTRC**)
- [x] Data Security
- [ ] Honest Broker
- [ ] UPMC Investigational Drug Service
- [ ] Pitt Medical School Review
- [x] Pitt+Me
- [ ] IND & IDE Support(**IIS**)
- [ ] Radioactive Drug Research Committee (**RDRC**)(study involves the evaluation or use of procedures that emit ionizing radiation)
- [ ] ORP Business **Manager** (required for industry sponsored studies)
- [ ] Religious Directives
- [ ] Scientific Review
- [ ] Health Record Research Request (**R3**) (required if using UPMC clinical data and authorization for other UPMC data sources for research)
- [ ] UPMC Office of Sponsored Programs and Research **Support** (using UPMC facilities and/or UPMC patients during the conduct of the study)

2. **Additional ancillary reviews the PI may choose to include as needed for the research:**

- [ ] Human Stem Cell Oversight (**hSCRO**)
- [ ] Institutional Biosafety Committee (**IBC**)(study involves deliberate transfer of recombinant or synthetic nucleic acid molecules)

View: Pitt SF: Clinical Trials

Good Clinical Practice (GCP) Training

1. * **Regardless of funding source, is this study a clinical trial (as defined by the NIH)?**
   ● **Yes** ○ No

ClinicalTrials.gov Information

Visit the University of Pittsburgh Office for ClinicalTrials.gov website or contact ctgov@pitt.edu for further information.

2. * **Was this study registered, or will it be registered, on ClinicalTrials.gov?**
   ● **Yes** ○ No

3. * **Is the University of Pittsburgh or UPMC the Sponsor Organization for this study record?**
   ● **Yes** ○ No

   * **Who will be the Responsible Party for this study record?**

   Principal Investigator of this IRB application

View: Pitt SF: Supporting Documents

Supporting Documents

1. **Attach any additional supporting documents not previously uploaded. Name the documents as you want them to appear in the approval letter:**

Document	Category	Date Modified	Document History

There are no items to display

View: Pitt Create

Add Storage Information

1. * **Select a Storage Type:**
   Other: Personal desktop, laptop, or other device

2. **Description:**
   Home Apple Desktop - Kim Rak

3. * **Will identifiable data be stored in this location?**
   ● **Yes** ○ No

4. * **Will sensitive data be stored in this location?**
   ○ Yes ● **No**

   * **Define your encryption methods:**
   - **BitLocker (Windows) (**Turn on device encryption**)**
   - **File Vault (Mac) (**Use FileVault to encrypt the startup disk on your Mac**)**
   - **SecureZIP (**SecureZIP: Getting Started (Windows) | University of Pittsburgh**)**

   File Vault - XTS-AES-128 encryption with a 256-bit key to help prevent unauthorized access to the information. Also has Antivirus provided by Malwarebytes or Sophos.

5. **Will de-Identified or anonymous data be stored in this location?**
   ○ Yes ● **No**

6. * **Is anti-virus software installed and up to date on all devices and are the operating systems kept up-to-date on all devices?**
   ● **Yes** ○ No

7. **Provide additional information as needed:**

View: Pitt Create

Add Storage Information

1. * **Select a Storage Type:**
   PITT: OneDrive / Sharepoint

2. **Description:**
   Critical Care Medicine Sharepoint Site

3. * **Will identifiable data be stored in this location?**
   ● **Yes** ○ No

   * **Describe how the data will be transferred, how the data be encrypted in transit and how the data be encrypted when stored:**
   Data will be transferred directly from Qualtrics or HRDC to PItt SharePoint if needed.

4. * **Will sensitive data be stored in this location?**
   ○ Yes ● **No**

5. **Will de-Identified or anonymous data be stored in this location?**
   ● **Yes** ○ No

6. **Provide additional information as needed:**

7. **Will access to the files or folders be restricted to only those research team members involved in the study(e.g., Specific people are granted access)?**
   ● **Yes** ○ No

View: Pitt Create

Add Storage Information

1. * **Select a Storage Type:**
   Server: Pitt Department Managed Server

2. **Description:**
   Data will be downloaded from the study website on to the HSRDC server and will be made available to the study team. Medicare claims data is also being obtained and will be stored on the HSRDC.

3. * **Will identifiable data be stored in this location?**
   ● **Yes** ○ No

4. * **Will sensitive data be stored in this location?**
   ○ Yes ● **No**

5. **Will de-Identified or anonymous data be stored in this location?**
   ● **Yes** ○ No

6. **Provide additional information as needed:**

View: Pitt Create

Add Storage Information

1. * **Select a Storage Type:**
PITT owned desktop, laptop or other device

2. **Description:**
Pitt Qualitative Research laptop* will be used to upload information from recorder to SharePoint

*PC Laptop with Bitlocker, which by default, uses the AES encryption algorithm in cipher block chaining (CBC) or XTS mode with a 128-bit or 256-bit key. University of Pittsburgh managed Antivirus.

3. * **Will identifiable data be stored in this location?**
○ Yes ● **No**

4. * **Will sensitive data be stored in this location?**
○ Yes ● **No**

5. **Will de-Identified or anonymous data be stored in this location?**
○ Yes ● **No**

6. * **Is anti-virus software installed and up to date on all devices and are the operating systems kept up-to-date on all devices?**
● **Yes** ○ No

7. **Provide additional information as needed:**

View: Pitt Create

Add Storage Information

1. * **Select a Storage Type:**
   Other: Cloud Storage Service other than Pitt or UPMC OneDrive

2. **Description:**
   Pitt Zoom

3. * **Will identifiable data be stored in this location?**
   ● **Yes** ○ No

   * **Describe how the data will be transferred, how the data be encrypted in transit and how the data be encrypted when stored:**
   Pitt Zoom will be used to conduct interviews and the resulting recorded files will be transferred to CCM SharePoint for storage

4. * **Will sensitive data be stored in this location?**
   ○ Yes ● **No**

5. **Will de-Identified or anonymous data be stored in this location?**
   ● **Yes** ○ No

6. **Provide additional information as needed:**

7. **Will access to the files or folders be restricted to only those research team members involved in the study(e.g., Specific people are granted access)?**
   ● **Yes** ○ No

View: Pitt Risk

Risk

1. * **Research Activity:**
   Use of video game and on-line assessment

2. **Common Risks:**
   None

3. **Infrequent Risks:**
   Lack of voluntariness, employability, confidentiality

4. **Other Risks:**
   None.
