# Supplementary material for: An Educational Video Game in Trauma Triage at Nontrauma Centers: A Secondary Analysis of a Randomized Clinical Trial
Source: JAMA Netw Open. 2025 Jun 4;8(6):e2513375. doi: 10.1001/jamanetworkopen.2025.13375 (PMC12138726; doi:10.1001/jamanetworkopen.2025.13375)
Supplement: Supplement 2. — eMethods. Intervention and Simulation Development, Physician Survey, Intention-to-Treat Principle, Signal Detection Theory Analysis, and Heterogeneity of Treatment Effect Analyses eTable 1. Physician Use of the Intervention eTable 2. Sensitivity Analysis to Test the Effect of Missing Outcome Data Using Inverse Probability Weighting eFigure 1. Schematic of the Conceptual Framework of the Intervention eFigure 2. Schematic of the Conceptual Framework of the Simulation eFigure 3. Association Among Clinical Workload, Gender, and Individualized Absolute Risk Reduction Estimates eFigure 4. Caterpillar Plot of individualized Absolute Risk Reduction Estimates From Bayesian Additive Regression Trees With 3 Predictors (Primary Model) eFigure 5. Caterpillar Plot of Individualized Absolute Risk Reduction Estimates From Bayesian Additive Regression Trees With 14 Predictors (Secondary Model) [file jamanetwopen-e2513375-s002.pdf]

Supplementary Online Content

Mohan D, Fischhoff B, Talisa V, et al. An educational video game in trauma triage at nontrauma centers: a secondary analysis of a randomized clinical trial. *JAMA Netw Open.* 2025;8(6):e2513375. doi:10.1001/jamanetworkopen.2025.13375

eMethods. Intervention and Simulation Development, Physician Survey, Intention-to-Treat Principle, Signal Detection Theory Analysis, and Heterogeneity of Treatment Effect Analyses
eTable 1. Physician Use of the Intervention
eTable 2. Sensitivity Analysis to Test the Effect of Missing Outcome Data Using Inverse Probability Weighting
eFigure 1. Schematic of the Conceptual Framework of the Intervention
eFigure 2. Schematic of the Conceptual Framework of the Simulation
eFigure 3. Association Among Clinical Workload, Gender, and Individualized Absolute Risk Reduction Estimates
eFigure 4. Caterpillar Plot of individualized Absolute Risk Reduction Estimates From Bayesian Additive Regression Trees With 3 Predictors (Primary Model)
eFigure 5. Caterpillar Plot of Individualized Absolute Risk Reduction Estimates From Bayesian Additive Regression Trees With 14 Predictors (Secondary Model)

This supplementary material has been provided by the authors to give readers additional information about their work.

© 2025 Mohan D et al. *JAMA Network Open*.

eMethods. Intervention and Simulation Development, Physician Survey, Intention-to-Treat Principle, Signal Detection Theory Analysis, and Heterogeneity of Treatment Effect Analyses

Intervention development

We followed a theory-based process that included: a) identifying heuristics as the potential cause of non-compliance with clinical practice guidelines in trauma triage; b) specifying narrative persuasion (i.e., compelling stories) as a theory of behavior change that we could use to align heuristics with clinical practice guidelines (i.e., to improve calibration); c) mapping narrative persuasion to behavior change techniques (e.g., shaping knowledge); d) selecting video games as the mode of intervention; and e) picking game mechanics that would deliver the behavior change techniques and also engage (i.e., immerse) the user in the experience. In preparation for the current trial, we refined the game based on user feedback, producing *Night Shift 2024*.

Players take on the persona of Andy Jordan, a young emergency medicine physician, who moves home to search for his missing grandfather, and takes a job at a small community hospital. The player must not only solve the mystery but also manage a series of trauma and non-trauma cases, experiencing the consequences of decision making. In the process, players receive feedback on their performance, during which in-game characters draw attention to the cues (e.g., the occurrence of injuries in of multiple body regions) that should inform the recognition of severely injured patients. Components of the game not directly advancing the learning goals were designed to stimulate engagement, including identification with the avatar, immersion, and transportation. We designed *Night Shift* to take approximately 2 hours to complete.

Simulation development

We had previously developed a bank of 100 cases, based on the case histories of patients admitted to a busy urban Level I trauma and critical care services, which we piloted in

© 2025 Mohan D et al. *JAMA Network Open*.

prior trials. We selected 60 cases for inclusion, balancing the case characteristics (e.g., injury mechanism, age of patients) to ensure a representative sample. At the start of the simulation, users had the opportunity to select the consultants they would ordinarily have available. We structured the simulation into 3 15-minute blocks, giving the player the option of taking a break between sessions. Among the set of cases evaluated by each user, 10 had non-traumatic complaints and were included to distract users,13 had severe injuries (mean Injury Severity Score [ISS] 18.5, range 16-29), and 13 had minor injuries (mean ISS 5.6, range 1-11).

We included several design elements to increase the verisimilitude of the experience. Patients arrived at pre-specified but unpredictable intervals, so that physicians had to manage multiple patients concurrently. Physicians could select the order in which they saw patients, based on their physiologic status as represented on a tracking board. New information returned in a time-delayed fashion, intended to replicate actual practice but scaled to the simulation. A clock at the top of the screen, allowed players to track the passage of time. Physicians could not make a disposition decision for hemodynamically unstable patients, to prevent them from deflecting responsibility for a case. In addition to their clinical responsibilities, players had to respond to audio-visual distractors that ranged from the alarms of medication pumps to paging alerts to nurses who would ask for help with patients in the waiting room. Finally, we programmed consultants to disagree with the physician's decision for the disposition of a patient in 50% of cases.

Physician survey

Each physician completed a questionnaire at the time of enrollment describing their age, gender, race, exposure to video games, educational background (board certification, years since completion of residency, ATLS certification, completion of the American Board of Emergency Medicine's maintenance of certification module on trauma resuscitation) and

© 2025 Mohan D et al. *JAMA Network Open*.

practice environment (trauma designation of their hospital, number of shifts they worked per month, region of the country).

Intention-to-treat (ITT) principle

Our primary analysis adhered to the ITT principle, with the following considerations: [a] randomization was preserved; [b] the probability of evaluating a severe injury case was independent of treatment assignment; [c] all randomized participants who encountered severe injury cases were included in the analysis (i.e., no post-randomization exclusion occurred due to case selection or missingness). To confirm that we met criteria [b], we applied the inverse probability weighting method. Specifically, we modeled the probability of a case being classified as a severe injury based on pre-randomization covariates, and derived weights based on the inverse of this probability. Finally, we fit generalized linear mixed model with binomial error and logit link functions to the weighted sample to obtain the estimated intervention effect. This approach allowed us to adjust for possible post-randomization selection and approximate the full-randomized population, thereby confirming our alignment with the ITT principle.

Signal detection theory analysis

With adequate trials (≥100), it is possible to estimate signal detection parameters directly from decisions. Where the cognitive burden of a task limits the number of trials, as it did here, it is possible to use a regression-based approach to estimate the parameters with confidence intervals. To estimate the signal detection theory parameters, we used the following equations.

$$Perceptual\ sensitivity\ (d') = logit(hit\ rate) - logit(false\ alarm\ rate)$$
$$= ln(hit\ rate) - ln(miss\ rate) - ln(false\ alarm\ rate) + ln(reject\ rate)$$
$$Decisional\ threshold\ (c) = -logit(false\ alarm\ rate)$$
$$= -ln(false\ alarm\ rate) + ln(reject\ rate)$$
$$logit\ p(Y = 1|X) = -c + d'X$$

© 2025 Mohan D et al. *JAMA Network Open*.

where Y is the disposition decision and X is the reference standard. The fit of logistic models approaches signal detection models, but with a scale $\pi / \sqrt{3}$ times larger than the Gaussian distribution used in signal detection theory. We fit a mixed effect model, predicting the log-odds of disposition decisions (dependent variable) using an intercept, the ACS-COT recommendation for the case and the intervention assignment of the physician (independent variables), clustered by physician. The model's intercept provided an estimate for the decisional threshold. A negative decisional threshold indicated a tendency to transfer trauma patients (i.e., effectively preferring false alarms to misses), and a positive value indicated a tendency not to transfer. The regression weight on the ACS-COT recommendation represented perceptual sensitivity, increasing with greater ability to discriminate between severely and minimally injured patients. We scaled our estimates by 1.8 to approximate the standard deviation units of signal detection values.

Heterogeneity of treatment effect analyses

We did not observe any missing values of provider clinical workload, age or gender. However, we did observe missing values in four other characteristics: race (N=64 missing, 7.3%), ethnicity (N=45 missing, 5.6%), number of years since completing ATLS (N=34 missing, 5.9%), and any experience working in a Level 1 or 2 trauma center (N=28 missing, 4.9%). Missing values for years since completion of ATLS were given the lowest value in an ordinal scale ranging from 0 (missing), 1 (<4 years ago) and 2 (<1 year ago). The other variables were imputed using mean imputation as follows: unknown race was imputed to be White; unknown ethnicity imputed to be non-Hispanic; and trauma center experience was imputed as "Yes."

Current practices for modeling of individualized absolute risk reduction (iARRs) suggest inclusion of baseline outcome risk, largely because good prediction of risk differences are mathematically dependent on baseline risk. Thus, we used random-intercept BART to model risk of under-triage among providers in the control group first, to understand whether our

© 2025 Mohan D et al. *JAMA Network Open*.

covariates were predictive of under-triage. Our model included as predictors 13 provider characteristics: age, race, ethnicity, gender, clinical workload, number of years since ATLS completion, trauma center experience, geographical region, trauma center designation of hospital of primary employment, way of learning about the trial, whether games were ever played for fun, fellowship completion, and type of residency. Provider ID was also included as a random intercept with an assumed normal distribution. This model achieved an AUC in the training sample of 0.63. We then fit a model among all providers that read at least one severely injured case, blind to their intervention group status, for inclusion into our model for iARR estimation. This method has been suggested to reduce bias. From this model, we estimated a single estimate of risk per provider, conditional only on provider characteristics, in the following way. For each of 1000 MCMC iterations (after 500 burn-in iterations) from the random-intercept probit BART model, we saved a single draw from the linear predictor for each provider and from the random intercept variance parameter. We then calculated, still for each MCMC iteration, the inverse probit of the sum of the linear predictor and a random draw from a normal distribution given the drawn variance parameter. This represented a posterior draw from the marginal distribution of the risk of under-triage. Finally, we took the median of this posterior for each provider as their predicted risk of under-triage.

We then estimated iARRs in two analysis models: 1) a model using only age, gender and clinical workload (primary model), and 2) a model using the 13 predictors in addition to the predicted baseline risk of under-triage blinded to treatment (secondary model). In both cases, iARRs were estimated as follows. A random intercept probit BART model of under-triage was fit using the appropriate covariate set, the intervention variable, as well as random intercept for provider as above. We ran 500 burn-in iterations and 1000 posterior draws from the MCMC chain. Using the parameter draws at each iteration, we predicted two linear predictors for each provider: one assuming the provider had been part of the intervention group, and another assuming they had been part of the control group. We then added to each of these predicted

© 2025 Mohan D et al. *JAMA Network Open*.

linear predictors a draw from the random intercept distribution (a normal distribution with variance using a draw from the appropriate posterior). Finally, still for each MCMC iteration, we took the inverse probit of each of these sums (linear predictor assuming for control arm + random effect, and linear predictor assuming intervention arm + random effect) for each provider, and took their difference. This difference was regarded as a draw from the posterior for the marginal iARR (conditional only on observed provider characteristics). We then summarized these posteriors by taking the median as the point estimate and the 2.5th and 97.5th percentiles as the 95% credible interval bounds. All random intercept probit BART modeling was performed using R package `dbarts`.

© 2025 Mohan D et al. *JAMA Network Open*.

ADDITIONAL RESULTS

eTable 1. Physician Use of the Intervention

	**Participants** (N=400)
Time spent playing Night Shift (n, %)	
None	2 (1)
≤60 min	22 (6)
61-120 min	141 (35)
>120 min	235 (59)
% of Night Shift completed (n, %)	
≤25%	16 (4)
26-50%	54 (14)
50-99%	62 (16)
100%	268 (67)

© 2025 Mohan D et al. *JAMA Network Open*.

eTable 2. Sensitivity Analysis to Test the Effect of Missing Outcome Data Using Inverse Probability Weighting

	Control	Intervention	Difference	95% Confidence Interval (CI)	p value
**Model 1 – benefit of intervention**					
Under-triage	63%	79%	16%	12 to 21%	<0.001

© 2025 Mohan D et al. *JAMA Network Open*.

eFIGURES

eFigure 1. Schematic of the Conceptual Framework of the Intervention We show the components of the intervention, the proposed mechanism of action, and the behavioral outcome we were hoping to achieve.

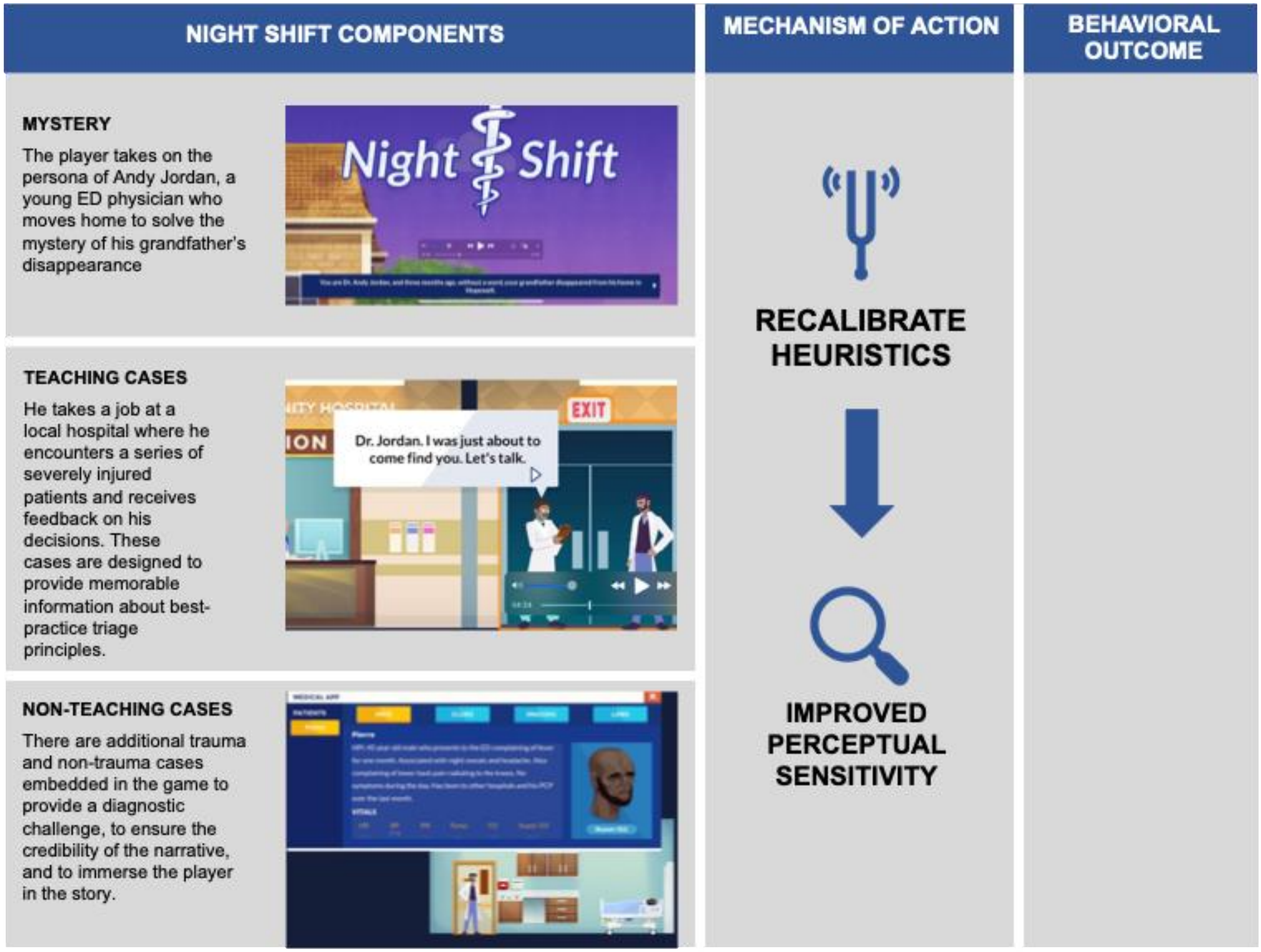

© 2025 Mohan D et al. *JAMA Network Open*.

eFigure 2. Schematic of the Conceptual Framework of the Simulation We show the structure of the simulation, the case format, and design choices that we made to increase verisimilitude.

SONAR COMPONENTS	SCREENSHOTS FROM SONAR
**Structure of simulation**<br>Users must complete 36 cases split across 3 15-minute 'shifts.' They have the option to pause between these shifts but must complete the simulation in one sitting. They can select the order in which they review cases within the block by using the control board.	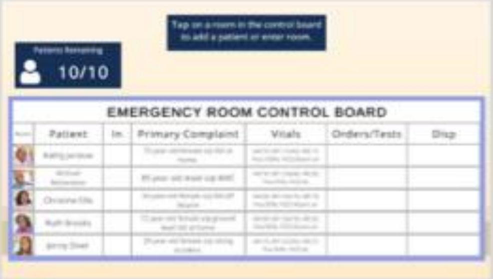
**Case format**<br>Users access information about each patient by reviewing case presentation data, physical exam findings, and by ordering diagnostic tests.<br><br>Cases have branching logic, so that severely injured or critically ill patients will decompensate if the user does not perform appropriate interventions (e.g., transfusing patients in hemorrhagic shock).	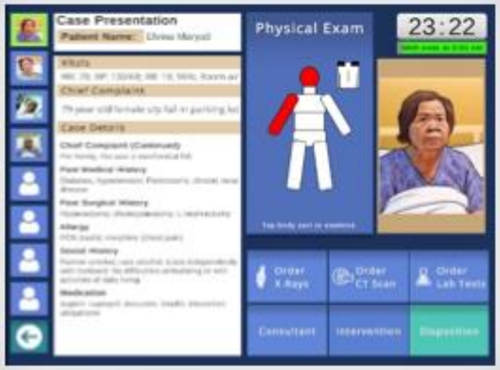
**Strategies to increase verisimilitude**<br>The simulation includes multiple audiovisual distractors (e.g., paging alerts, IV poles beeping). In this example, the nurse interrupts the user to ask for help managing a busy waiting room. Additional strategies to increase the realism of the experience include the need to manage multiple patients concurrently and to review data added to patients' charts as tests occur.	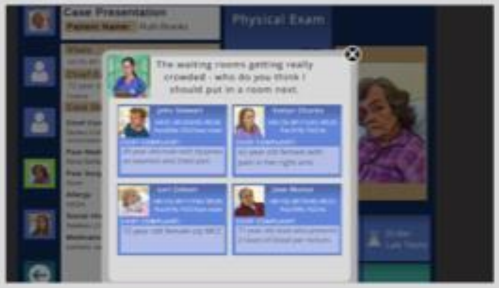

© 2025 Mohan D et al. *JAMA Network Open*.

eFigure 3. Association Among Clinical Workload, Gender, and Individualized Absolute Risk Reduction Estimates

An inflection point appears to occur at 10 shifts per month with physicians working ≥10 shifts experiencing a greater effect than those who work less than 10 shifts per month. The model also shows that men (showed with black circles) experience a greater effect than women (showed with red circles).

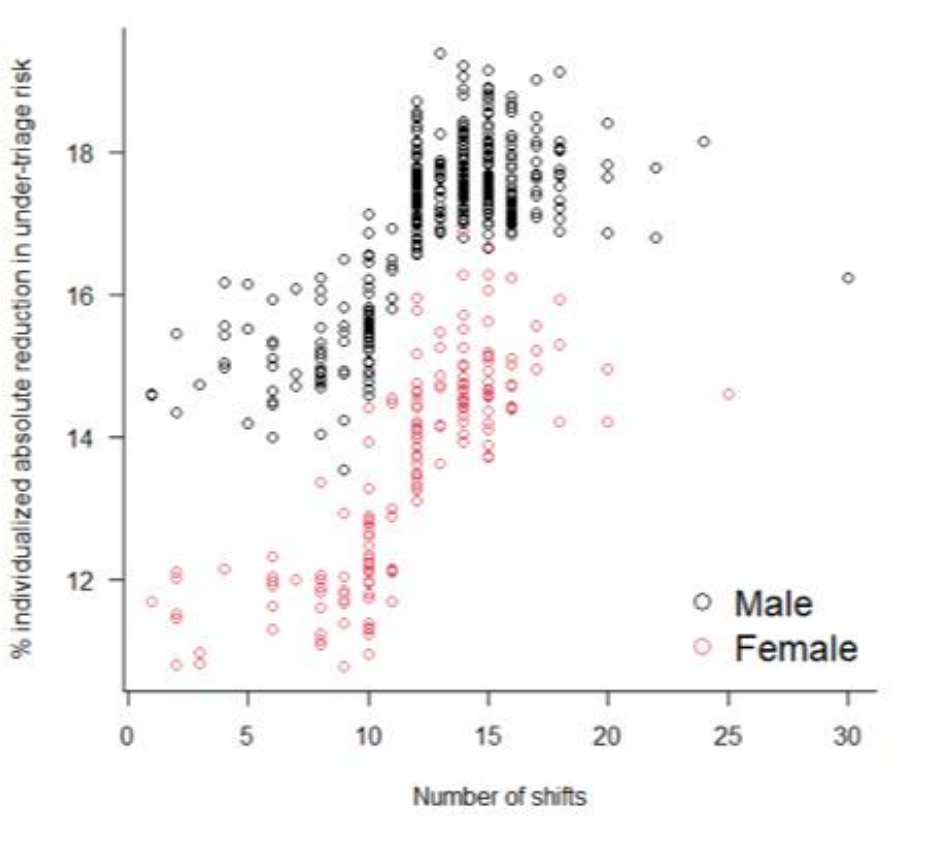

© 2025 Mohan D et al. *JAMA Network Open*.

eFigure 4. Caterpillar Plot of individualized Absolute Risk Reduction Estimates From Bayesian Additive Regression Trees With 3 Predictors (Primary Model)

We show the estimates, along with 95% credible intervals, from models trained on provider age, gender, and clinical workload. The effect size ranged from 10.1% to 19.6%. The dashed horizontal line indicates the null estimated effect of intervention over control, while the dotted horizontal line indicates a clinically meaningful effect of 10% individualized reduction in risk due to intervention.

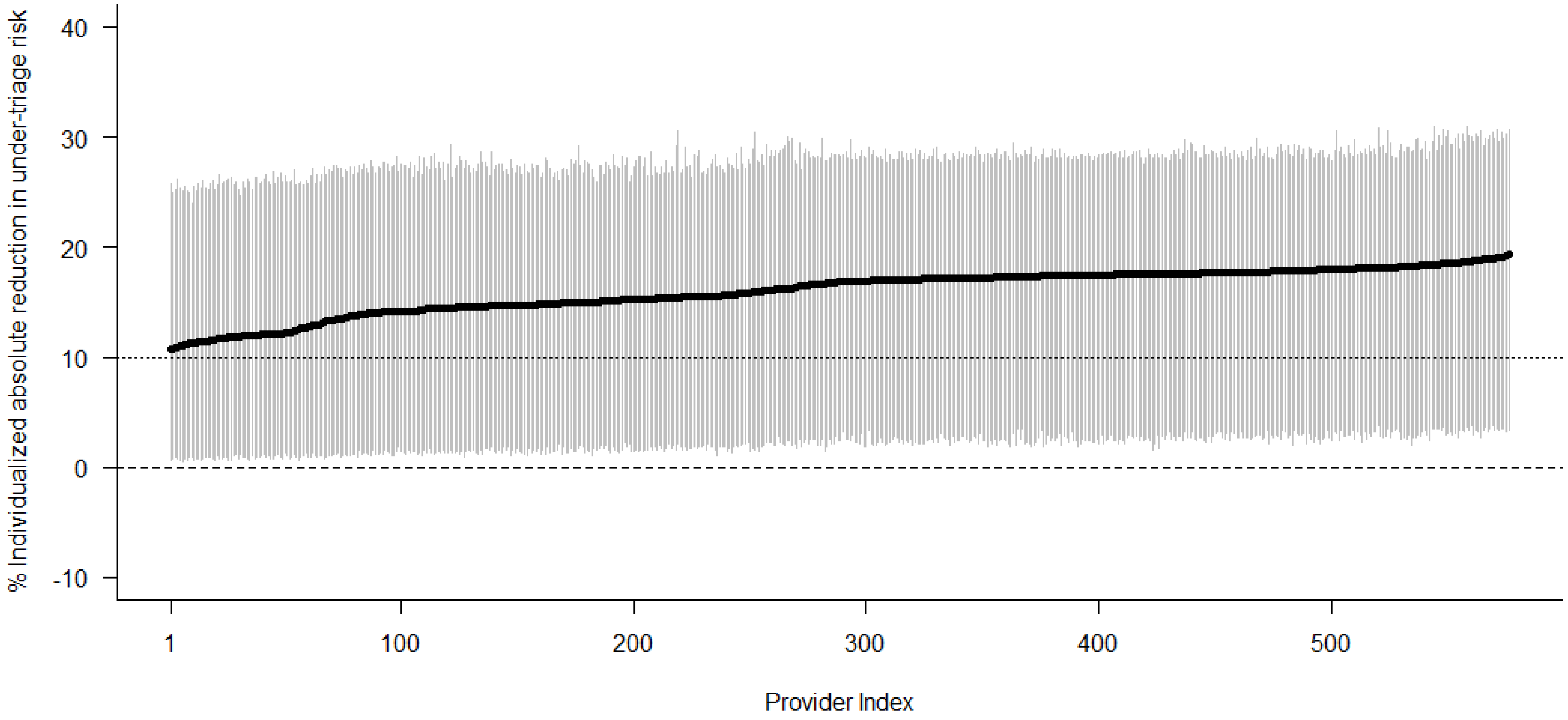

© 2025 Mohan D et al. *JAMA Network Open*.

eFigure 5. Caterpillar Plot of Individualized Absolute Risk Reduction Estimates From Bayesian Additive Regression Trees With 14 Predictors (Secondary Model)

We show the estimates, along with 95% credible intervals, from models trained on 13 physician characteristics along with estimated baseline risk of under-triage. The effect size ranged from 8.4% to 16.0%.

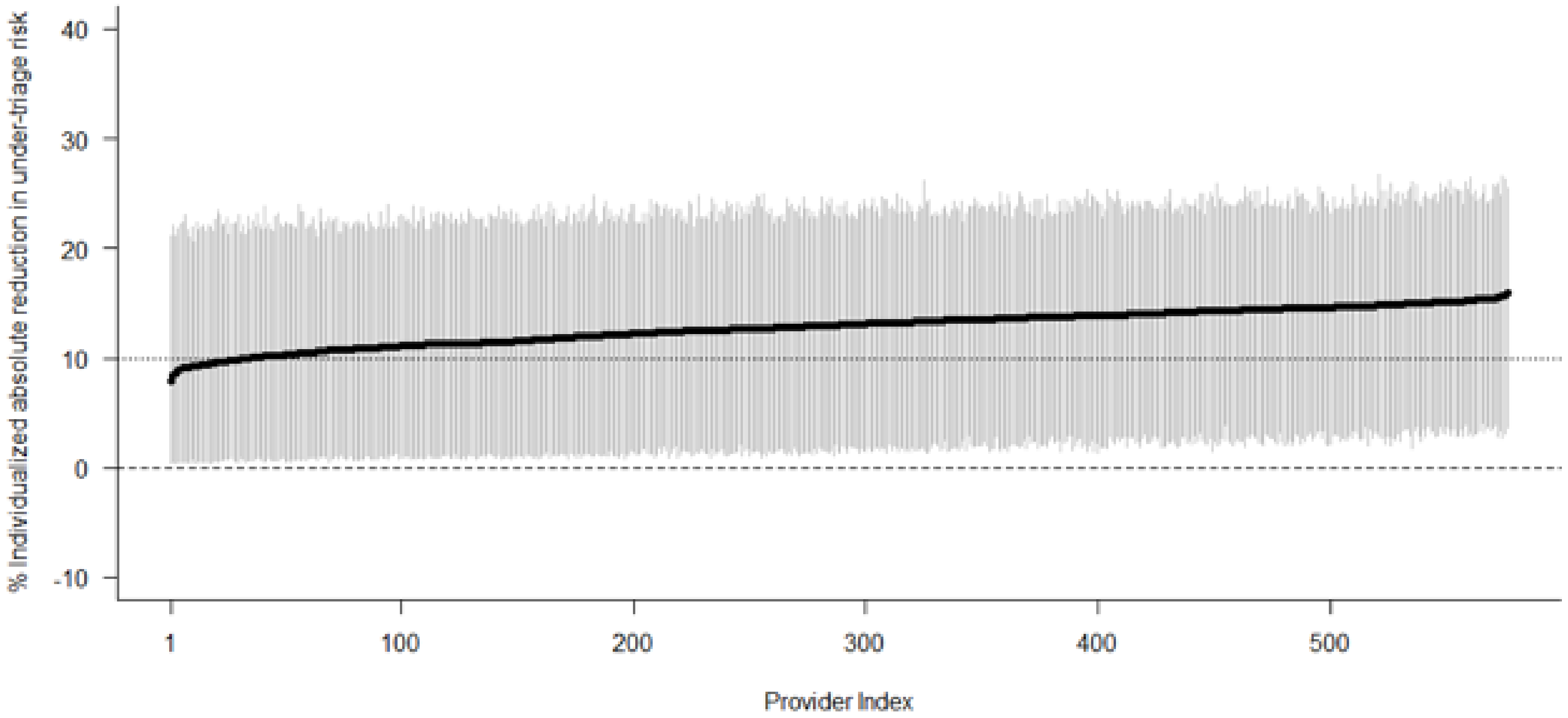

© 2025 Mohan D et al. *JAMA Network Open*.
