# Supplementary material for: An Educational Video Game in Trauma Triage at Nontrauma Centers: A Secondary Analysis of a Randomized Clinical Trial
Source: JAMA Netw Open. 2025 Jun 4;8(6):e2513375. doi: 10.1001/jamanetworkopen.2025.13375 (PMC12138726; doi:10.1001/jamanetworkopen.2025.13375)
Supplement: Supplement 3. — Data Sharing Statement [file jamanetwopen-e2513375-s003.pdf]

Data Sharing Statement

Mohan. An Educational Video Game in Trauma Triage at Nontrauma Centers. *JAMA Netw Open*. Published June 04, 2025. doi:10.1001/jamanetworkopen.2025.13375

Data
Additional Information: ClinicalTrials.gov; NCT06063434. Registered 26 September 2023, https://classic.clinicaltrials.gov/ct2/show/NCT06063434
Data available: Yes
Data types: Deidentified participant data
How to access data: mohand@upmc.edu
When available: With publication

Supporting Documents
Document types: Other (please specify)
Additional Information: Data dictionary
How to access documents: mohand@upmc.edu
When available: With publication

Additional Information
Who can access the data: Researchers whose proposed use of the data has been approved
Types of analyses: Any purpose approved by the University of Pittsburgh Office of Research
Mechanisms of data availability: Signed data use agreement
